# Open Anterior Mesh Repair vs Modified Open Anterior Mesh Repair for Groin Hernia in Women: A Randomized Clinical Trial

**DOI:** 10.1001/jamasurg.2025.2244

**Published:** 2025-07-16

**Authors:** Alphonsus Matovu, Pär Nordin, Andreas Wladis, Gabriel Sandblom, Moses Elaju, Fredrik Lindmark, Olof Bladin, Jenny Löfgren

**Affiliations:** 1 Department of Surgery, Mubende Regional Referral Hospital, Mubende, Uganda; 2 Department of Molecular Medicine and Surgery, Karolinska Institutet, Stockholm, Sweden; 3 Department of Diagnostics and Intervention/Surgery Department of Östersund, Umeå University, Umeå, Sweden; 4 Department of Biomedical and Clinical Sciences, Linköping University, Linköping, Sweden; 5 Department of Surgery, Södersjukhuset, Stockholm, Sweden; 6 Department of Clinical Science and Education Södersjukhuset, Karolinska Institutet, Solna, Sweden; 7 Department of Surgery, Mbale Regional Referral Hospital, Mbale, Uganda; 8 Department of Surgery, Östersund Hospital, Östersund, Sweden; 9 Department of Reconstructive Plastic Surgery, Karolinska University Hospital, Stockholm, Sweden

## Abstract

**Importance:**

Most women in low- and middle-income countries lack access to laparoscopic methods for groin hernia repair; therefore, an open technique through which both inguinal and femoral hernias can be treated is needed. This could be an option in the absence or inability to use laparoscopic methods.

**Objective:**

To determine the safety and effectiveness of open anterior mesh (OAM) repair compared with modified open anterior mesh (MOAM) repair, which includes opening the transversalis fascia and covering the femoral canal with a mesh flap.

**Design, Setting, and Participants:**

This was a parallel, 2-arm, double-blind, randomized clinical trial conducted in Northern Uganda, in East Africa, at 2 public hospitals between October 2019 and February 2023. Included in the study were adult women 18 years and older with a primary groin hernia, American Society of Anesthesiologists (ASA) class I or II, and the ability to give informed consent.

**Interventions:**

OAM in the control arm and MOAM in the intervention arm.

**Main Outcomes and Measures:**

The primary outcome was groin hernia recurrence 1 year postoperatively.

**Results:**

A total of 200 participants (mean [SD] age, 52.7 [14.0] years) were included in the study; 99 (49.5%) were allocated to OAM repair, and 101 (50.5%) were allocated to MOAM repair. Nearly 45% of the participants (89 of 200) had a femoral hernia; therefore, 35 of 99 participants (35.4%) in the control arm received the intervention procedure. One year postoperatively, the overall recurrence was 5.6% (11 of 195 participants), and the intention-to-treat analysis showed that 4 of 97 participants (4.1%) in the control arm and 7 of 98 participants (7.1%) in the intervention arm had recurrence (absolute difference = −3.0 percentage points; 95% CI, −9.5 to 3.4; *P* = .36).

**Conclusions and Relevance:**

Results of this randomized clinical trial demonstrate that the MOAM repair was a good option for groin hernia repair in women in low-resource settings. Femoral hernias were very common in the study population, and exposure of the femoral canal was essential to detect these hernias.

**Trial Registration:**

ISRCTN Identifier: ISRCTN10330683

## Key Points

**Question:**

What is the safety and effectiveness of open anterior mesh repair compared with modified open anterior mesh repair with a mesh flap covering the femoral canal for groin hernias among women in a low-income setting?

**Findings:**

In this randomized clinical trial including 200 women with a primary groin hernia, approximately half had femoral hernias. One year postoperatively, the recurrence rate was similar in the 2 study groups.

**Meaning:**

Results demonstrate that the modified open anterior mesh repair can treat both inguinal and femoral hernias in women, but further evaluation is warranted.

## Teaser

This randomized clinical trial investigates the safety and effectiveness of open anterior mesh repair compared with modified open anterior mesh repair for groin hernias among women in a low-income setting.

## Introduction

Groin hernia is a common surgical condition, and effective treatment is by surgical repair.^1^ Previous estimates show that over 200 million people have the condition with 20 million repairs annually, 10% in women.^2^

Surgical methods treating groin hernias have evolved to improve effectiveness and quality of care.^3,4^ For example, shifting from tissue repairs to tension-free repairs with mesh reduced recurrence rates by nearly 50% in men.^5,6^ In addition, substituting open for laparoscopic approaches has been adopted to improve outcomes including recurrence and postoperative pain.^7,8,9^ Women more frequently than men experience groin hernia recurrence after open repair. This could possibly be a result of missed femoral hernias. Thereby, laparoscopic approaches are recommended enabling visualization of both inguinal and femoral hernias.^10,11^ However, the high costs involved limit the availability of laparoscopic approaches in low- and middle-income countries (LMICs), where two-thirds of the world’s population live.^12,13^ Effective and safe open surgical techniques are therefore needed.

In LMICs such as Uganda, groin hernia repair in women is mostly performed using suture techniques.^14^ To improve outcomes, an open anterior mesh (OAM) repair suitable for inguinal and femoral hernias is needed.

The present aim was to compare safety and effectiveness of OAM repair according to Lichtenstein with modified open anterior mesh (MOAM) repair through which femoral hernias can be identified and repaired.

## Methods

### Study Design

This was a parallel, 2-arm, double-blinded, randomized clinical trial (RCT) comparing the effectiveness of OAM repair (control arm) with MOAM repair (intervention arm) for groin hernia repair in adult women in Uganda. The study was carried out and analyzed according to the Consolidated Standards of Reporting Trials (CONSORT) reporting guidelines. Ethical approval was obtained from Mildmay Uganda Research and Ethics Committee (MUREC) and the Uganda National Council of Science and Technology. The original trial protocol and statistical analysis plan are available in Supplement 1 and Supplement 2, respectively.

### Study Participants

African Ugandan women 18 years and older, with primary reducible groin hernia, American Society of Anesthesiologists Score (ASA) class I or II, and the ability to give informed consent were eligible study participants. Exclusion criteria were recurrent groin hernia, incarcerated groin hernia needing emergency surgery, known pregnancy, known bleeding disorders, and obvious alcohol or substance misuse.

### Randomization and Masking

A computer-based system was used to generate a randomization sequence of the treatment groups in blocks of 4, 6, and 8.^15^ Thereafter, the allocation arm for each study participant, in sequential order, was written on identical white paper and sealed in opaque envelopes. The ratio of randomization to the 2 study arms was 1:1. The day before surgery, the operation list for the participants was determined to guide entry to the operating room. The candidates were randomized by an independent person after the surgeon and the patient had entered the operating room. Thereafter, the surgeon was informed which method to use. A different surgeon (A.M.) did the follow-up 2 weeks and 1 year postoperatively. The outcome assessors and the participants were blinded to the treatment arms.

### Study Setting

The study was carried out in Uganda, a low-income country in Eastern sub-Saharan Africa with a population of nearly 46 million people.^16^ Screening, recruitment, and the surgical procedures were carried out at 2 public hospitals: Kitgum General Hospital and Arua Regional Referral Hospital in Northern Uganda, located 281 and 375 mi, respectively, from the capital city, Kampala. Women with suspected groin hernias were invited via radio announcements from several districts around the study hospitals. The same surgical team carried out the surgical procedures in the 2 hospitals.

### Data Collection and Follow-Up

Data were collected and entered in case report forms at 5 different stages: preoperatively, immediately after the surgical procedure, at discharge from the hospital, and 2 weeks and 1 year postoperatively. Preoperative data included demographic information and medical history. Preoperative physical examination was carried out by the surgeons on the team to ascertain the presence of a groin hernia. Ultrasound was used in 2 participants to confirm a groin hernia. Immediately after the surgical procedure, the surgeon filled out the form with intraoperative details regarding hernia anatomy and the surgical procedure. The principal investigator together with the research assistants filled out the data forms for the 1-week and 1-year follow-ups.

### Surgical Methods and Materials

All surgical procedures were performed by 4 surgical specialists with experience in groin hernia surgery using the same method for OAM repair and MOAM repair under local anesthesia.^17,18^ Before study start, the surgeons operated on 11 patients together until the team was satisfied that all surgeons were performing the surgical techniques the same way. Skin was prepared using povidone iodine, and local anesthesia consisted of an equal mix of lidocaine (10 mg/mL) and ropivacaine (7.5 mg/mL). A prophylactic antibiotic, oral clindamycin, in the first 82 participants and Bactrim Forte, a combination of sulfamethoxazole, 800 mg, and trimethoprim, 160 mg (Eumedica Pharmaceuticals GmbH) in the last 118 participants, was administered 1 hour before surgery. One oral dose of 1 g of paracetamol was administered together with the antibiotics. All procedures were performed under local anesthesia and most as outpatient surgery. Participants who underwent surgery late in the evening and those with postoperative bleeding from wound edges or where pain control was not achieved were admitted for overnight stay. A lightweight commercial flat mesh, Parietene (Medtronic), 15 cm × 10 cm, made of monofilament polypropylene was used in all patients.

In the control group, OAM repair according to Lichtenstein was used.^17^ In order not to miss femoral hernias, the transversalis fascia was opened to explore the femoral canal for a femoral hernia. If there was no femoral hernia, the opening in the transversalis fascia was closed with a continuous 2.0 polypropylene suture, and the anterior mesh repair was performed. If a femoral hernia was found, the patient received the MOAM repair described subsequently.

The participants in the intervention arm received MOAM repair as performed in an earlier pilot study basing on the Lichtenstein method.^19,20^ It includes all steps of OAM repair. In addition, after opening of the posterior wall (transversalis fascia) of the inguinal canal, an approximately 3-cm incision was made from the pubic tubercle laterally along the iliopubic tract parallel to the inguinal ligament. This gave access to the preperitoneal space and the femoral canal. If there was a femoral hernia or weakening, it was inspected and palpated above the Cooper ligament. If found, the hernia was reduced and invaginated under direct vision. The mesh was then fashioned on the lower edge to create a flap that could be extended to cover the femoral canal. The size of the flap was approximately 4 cm long and 3 cm wide. The lower part of this flap was fixed to the Cooper ligament with 2 nonabsorbable 2.0 polypropylene sutures, one medially beside the pubic tubercle and the other more laterally but at a safe distance from the femoral vessels. The incision in the posterior wall was closed by suturing the transversalis fascia to the mesh, ie, the upper part of the flap, followed by the final steps of a normal OAM repair according to Lichtenstein.^17^

### Outcomes

The primary outcome was groin hernia recurrence 1 year postoperatively. This was defined as presence of a reducible or irreducible bulge in the operated groin, assessed by A.M. Some cases were cross-checked by authors P.N. and J.L. Ultrasound was used in 3 participants to confirm recurrence. Secondary outcomes were assessed at 2 weeks and 1 year postoperatively. At 2 weeks postoperatively, the secondary outcomes were wound complications according to the Clavien-Dindo classification, pain according to the Inguinal Pain Questionnaire (IPQ), and patient satisfaction with the procedure.^21,22^ At 1 year postoperatively, the secondary outcomes were chronic pain according to the IPQ, satisfaction with the procedure, and mean health score for current overall health status.

### Statistical Analysis

The trial had a superiority study design assuming a recurrence of 6% in the control arm and 1% in the intervention arm. This assumption was based on a previous RCT on elective OAM repair among men in Uganda.^23^ Although not ideal, it was the best available evidence at the time. With 80% power and 5% precision rate, and an expected difference in recurrence rate of 5%, the expected success rate was 99% in the intervention arm after 1 year and 94% in the control arm, giving a sample size of 418 study participants. To compensate for unexpected loss to follow-up of 5%, the study planned to recruit 440 participants with 220 participants in each study arm.

Primary analysis was done according to the intention-to-treat principle. Per-protocol analysis was also carried out for comparison. Continuous variables were calculated as mean and SD. Binary variables were calculated as numbers and percentages. For comparison between the 2 study arms, a χ^2^ test was used for binary outcomes and a 2-sample *t* test for continuous variables. The 95% CI of the percentage points from the absolute differences and a *P* value were determined using independent sample proportions for binary outcomes and independent sample *t*-tests for continuous outcomes. SPSS statistical software, version 29.0.1.0 (IBM Corp) was used for all data analyses. All *P* values were 2-sided, and *P* <.05 was considered statistically significant. All statistical analyses were performed by A.M.

#### Changes to the Study Protocol

Three important alterations to the study protocol were made after the study start. The prophylactic antibiotic was changed in the second phase of recruitment after some of the participants in the first phase of recruitment reported experiencing diarrhea after hospital stay (amendment February 2021). The second alteration was opening of the transversalis fascia in the control arm as recommended in the International Hernia Surgery guidelines. This practice was introduced after the first 82 operations as the study team noted that the number of detected femoral hernias in the intervention arm appeared to be higher than in the control arm, indicating that femoral hernias may be missed unless the femoral canal is visualized (approved protocol 2020). These 2 alterations were approved by MUREC. The third alteration was completion of the study after the first study participants had been included, operated on, and followed up 1 year postoperatively. Due to the COVID-19 pandemic, the remaining participants could not be included in the study schedule. Therefore, 1-year follow-up was completed for a portion of participants before completion of the study’s inclusion phase. At the 1-year follow-up, the number of recurrences was higher than anticipated, which warranted further investigation before inclusion of additional study participants. The findings differed considerably from prestudy assumptions, and we concluded that complete data analysis was required. The approval to end the study early was obtained through an application for amendment to MUREC (amendment August 2024).

## Results

Altogether, 200 study participants (mean [SD] age, 52.7 [14.0] years) were included and operated on at the 2 study hospitals between October 8, 2019, and March 19, 2020 (Figure); 99 (49.5%) were allocated to OAM repair, and 101 (50.5%) were allocated to MOAM repair. The baseline characteristics for the participants in both arms were similar (Table 1). The mean (SD) duration for groin hernia repair in the control arm was 47.5 (13.0) minutes compared with 51.6 (13.9) minutes in the intervention arm. The intraoperative findings are presented in eTable 1 in Supplement 3. Overall, 89 participants (almost 45%) had femoral hernias; therefore, 35 of 99 participants (35.4%) in the control arm received the intervention procedure.

**Figure.**
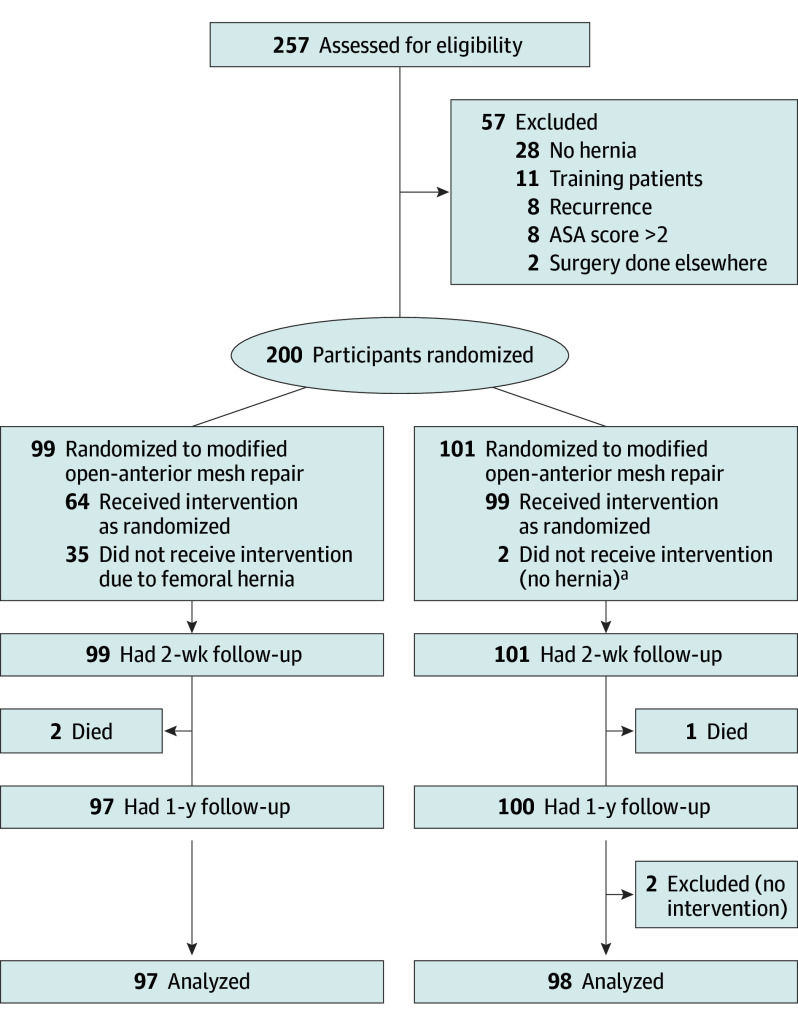
Consolidated Standards of Reporting Trials Flowchart Of the 257 participants assessed for eligibility, 200 were randomized into the study. These were allocated to either study arm. One hundred percent of the participants had both a 2-week to 1-year follow-up visit. ASA indicates American Society of Anesthesiologists. ^a^No hernia present; 1 had a Spigelian hernia and mesh was placed under the muscle, and 1 had no hernia and no mesh was placed.

**Table 1.** Baseline Characteristics of the Study Participants Stratified According to Allocation Arm in Intention-to-Treat and Per-Protocol Analyses^a^

Characteristics	Participants, No. (%)			
	Intention to treat		Per protocol	
	Open anterior mesh repair (n = 99)	Modified open anterior mesh repair (n = 99)	Open anterior mesh repair (n = 64)	Modified open anterior mesh repair (n = 134)
Age, mean (SD), y	51.7 (14.2)	53.9 (13.4)	52.0 (14.2)	53.2 (13.7)
BMI, mean (SD)^b^	20.1 (2.6)	19.8 (3.2)	20.3 (2.7)	19.8 (2.9)
ASA classification score of I	87 (87.9)	87 (87.9)	57 (89.1)	117 (87.3)
History of smoking	9 (9)	3 (3)	4 (6.3)	8 (5.9)
No. of deliveries, mean (SD)	6.5 (2.5)	6.4 (2.7)	6.4 (2.6)	6.4 (2.5)
History of cesarean delivery	9 (9)	0	7 (10.9)	2 (1.4)
Preoperative IPQ score, mean (SD)	3.5 (1.1)	3.4 (1.1)	3.6 (1.2)	3.4 (0.9)
Duration of pain >6 mo	91 (91.9)	92 (92.9)	56 (87.5)	127 (76.9)
Preoperative experience of severe groin pain	93 (93.9)	93 (93.9)	62 (96.8)	124 (92.5)
Self-Assessed Health Score, mean (SD)	58.1 (12.1)	55.9 (14.3)	56.5 (11.3)	57.3 (14.2)

Abbreviations: ASA, American Society of Anesthesiologists; BMI, body mass index; IPQ, inguinal pain questionnaire.

^a^ The analysis for this table is for 198 participants. Two participants were excluded from the analysis because they did not have any of the interventions.

^b^ Calculated as weight in kilograms divided by height in meters squared.

One year postoperatively, groin hernia recurrence was observed in 11 of 195 participants (5.6%) (Table 2). The recurrence rate was 4.1% (4 of 97 participants) in the control arm and 7.1% (7 of 98 participants) in the intervention arm. The absolute difference between the arms was −3.0 percentage points (95% CI, −9.5 to 3.4; *P* = .36). An open posterior approach according to Nyhus was used to repair recurrent hernias, and the findings are indicated in eTable 2 in Supplement 3. Three participants had died before 1-year follow-up: 2 in the control arm and 1 in the intervention arm. Information concerning their deaths was obtained from family members, and none appeared to be related to the surgical operation (eAppendix in Supplement 3).

**Table 2.** Primary Outcome Measure, Hernia Recurrence, and Death Among Study Participants Stratified by Allocation Arm in the Intention-to-Treat Analysis^a^

Outcome	Participants, No./total No. (%)		Absolute difference, percentage points (95% CI)	*P* value
	Open anterior mesh repair	Modified open anterior mesh repair		
Recurrence	4/97 (4.1)	7/98 (7.1)	−3.0 (−9.5 to 3.4)	.36
Death	2/99 (2.0)	1/99 (1.0)	1.0 (−2.4 to 4.4)	.56

^a^ The analysis for recurrence in this table is for 195 participants. Two participants were excluded from the analysis because they did not have any of the interventions, and 3 participants had died by the time of the 1-year follow-up, leaving 195 participants for the analysis.

The 2-week follow-up was conducted for all participants with interviews and physical examination. Wound complications were reported in 20 participants (10.1%), 12 (12.1%) in the control arm and 8 (8.1%) in the intervention arm (absolute difference = 4.0 percentage points; 95% CI, −4.3 to 12.4; *P* = .33) (Table 3). In total, 7 participants (3.5%) had impaired wound healing managed with wound dressing, 6 (3.0%) had superficial wound infections managed with antibiotics, and 5 (2.5%) had severe pain requiring extra analgesics. One participant with a deep infection was reoperated on to drain pus due to an infection, but the mesh was not removed. One participant with postoperative bleeding had 2 small vessels ligated in the operating room.

**Table 3.** Secondary Outcome Measures at 2 Weeks Stratified by Allocation Arm in the Intention-to-Treat Analysis^a^

Outcomes	Participant, No (%)		Absolute difference, percentage points (95% CI)	*P* value
	Anterior mesh repair (n = 99)	Modified open anterior mesh repair (n = 99)		
Postoperative wound complications, No. (%)	12 (12.1)	8 (8.1)	4.0 (−4.3 to 12.4)	.33
Severe pain needing extra medications	3 (3.0)	2 (2.0)	1.0 (−3.4 to 5.4)	.65
Bleeding requiring surgical intervention	1 (1.0)	0	1.0 (−1.0 to 3.0)	.32
Wound infection treated with antibiotics	5 (5.1)	1 (1.0)	4.1 (−1.0 to 9.0)	.97
Wound infection needing surgical intervention	1 (1.0)	0	1.0 (−1.0 to 3.0)	.32
Impaired wound healing	2 (2.0)	5 (5.0)	−3.0 (−8.2 to 2.1)	.25

^a^ The analysis for this table is for 198 participants. Two participants were excluded from the analysis because they did not have any of the interventions.

The 1-year mean (SD) follow-up time was 1.81 (0.04) years in both arms. The majority of the participants reported less groin symptoms than before the operation (92 of 97 [94.8%] and 93 of 98 [94.9%] in the control and intervention arms, respectively). Overall, the mean (SD) IPQ at 1 year was 1.53 (1.1) and 1.56 (0.94) in the control and intervention arms, respectively (absolute difference = −0.03; (95% CI, −3.2 to 2.5; *P* = .81). The mean (SD) IPQ difference (Δ IPQ) between baseline and 1 year postoperatively was 1.96 (1.5) and 1.85 (1.2) in the control and intervention arms, respectively (absolute difference = 0.11; 95% CI, −2.7 to 5.0; *P* = .56); however, 1 participant still reported severe pain (IPQ score = 7) at 1-year follow-up. Participants with severe pain were reviewed a second time to differentiate more thoroughly between pain due to hernia and other possible causes of chronic groin pain. This was done by A.M., P.N., and J.L. It was concluded that most complaints presenting as groin pain were most likely musculoskeletal in origin.

Ninety of 97 participants in the control arm (92.8%) and 93 of 98 participants (94.9%) in the intervention arm were satisfied with the outcome of the operation. The health thermometer at 1 year was used in 130 participants, giving mean (SD) scores of 87.4 (12.8) and 86.8 (14.8) in the control and intervention arms, respectively, and showing an absolute difference of 0.6 (95% CI, −4.0 to 5.3; *P* = .78). The mean (SD) difference in the health thermometer at 1 year vs baseline (Δ health score) was +30.2 (13.6) and +32.3 (16.1; absolute difference = −2.1; 95% CI, −7.3 to 3.1; *P* = .42) (Table 4). Per-protocol analysis was also carried out for comparison as indicated in Table 1 and eFigure and eTable 3 in Supplement 3.

**Table 4.** Secondary Outcome Measure at 1 Year in the Intention-to-Treat Analysis^a^

Outcome/variable	Participants, No (%)		Absolute difference, percentage points (95% CI)	*P* value
	Open anterior mesh repair (n = 97)	Modified open anterior mesh repair (n = 98)		
Less groin symptoms than before groin hernia repair	92 (94.8)	93 (94.9)	−0.1 (−6.1 to 6.2)	.99
Same groin symptoms as before operation	3/97 (3.0)	3/98 (3.1)	−0.1 (−5.0 to 5.0)	.99
More groin symptoms than before operation	2/97 (2.1)	2/98 (2.0)	0.1 (−4.0 to 4.0)	.99
Satisfied with the results of surgery 195 participants	90 (90.9)	93 (93.9)	−3 (−4.6 to 8.9)	.54
IPQ, mean (SD)	1.53 (1.1)	1.56 (0.9)	−0.03 (- 3.2 to 2.5)	.81
IPQ difference, mean (SD)	1.96 (1.5)	1.85 (1.2)	0.11 (−2.7 to 5.0)	.56
IPQ 1	72 (74.2)	67 (68.4)	5.8 (−6.8 to 18.5)	.37
IPQ 2-3	18 (18.6)	24 (24.5)	−5.9 (−17.4 to 5.6)	.31
IPQ 4-5	6 (6.2)	7 (7.1)	−0.9 (−8.0 to 6.0)	.79
IPQ >6	1 (1.0)	0 (0)	1.0 (−1.0 to 3.0)	.31
Health thermometer self-assessed health status, mean (SD)^b^	87.4 (12.8)	86.8 (14.2)	0.6 (−4.0 to 5.3)	.78
Difference in health status score at 1 year and baseline, mean (SD)^c^	30.2 (13.6)	32.3 (16.1)	−2.1 (7.3 to 3.1)	.42

Abbreviation: IPQ, inguinal pain questionnaire.

^a^ The analysis for this table is for 195 participants. Two participants were excluded from the analysis because they did not have any of the interventions, and 3 participants had died by the time of the 1 year follow-up.

^b^ For 130 participants.

^c^ For 130 participants.

## Discussion

In this RCT of 200 women, the 1-year groin hernia recurrence rate was 5.6%, and postoperative complications occurred in 10.1%. Almost half of the study participants had femoral hernias.

The overall groin hernia recurrence rate of 5.6%, of which 4.1% occurred in the control arm and 7.1% in the intervention arm, was higher than expected by the study team. However, the study did not show superiority of any technique. To our knowledge, no previous RCTs evaluating surgical techniques for OAM repair in women exist for comparison with our results. However, a previous systematic review reported a recurrence rate of 4.9% after OAM repair in women, and this is similar to our results.^24^ After AOM repair, femoral hernias have a higher risk of recurrence compared with inguinal hernias, ranging between 20% and 50%; in particular if the femoral canal is not explored.^25,26^ Previous studies that have reported using mesh to cover the femoral canal do not report any recurrences after long follow-up periods. However, these studies were small, and procedures were performed by 1 surgeon.^27,28^

The postoperative complication rates were almost the same in the 2 groups, with an overall rate of 10.1%. These were lower than in similar studies carried out in adult men (30.2% in Uganda, 26.6% in Ghana, and 29.7% in Sierra Leone).^23,29,30^ By exploring the preperitoneal space, we expanded the field of surgery but with no apparent increase in complications. The study further corroborates that using mesh in the setting of LMICs is safe for elective groin hernia surgery in women as well as in men.^31,32^

In previous studies, the proportion of femoral hernias in women ranges between 16.7% and 37%.^33,34^ Before the transversalis fascia was opened in all our study participants, the proportion of femoral hernias identified in the control arm was lower than that in the intervention arm, indicating that some femoral hernias could have been undetected. The large number of femoral hernias in the present study resulted in 35 of the participants (17.5%) allocated to the control arm receiving the intervention procedure. The high crossovers disadvantage the OAM repair in populations with a high prevalence of femoral hernias. It is probable that many femoral hernias are routinely missed in the study setting because opening of the transversalis fascia is not routine. Because women face a higher risk of a recurrence and a hernia emergency, due to femoral hernias, it is highly recommended to always visualize the femoral canal during this surgery in women.

Chronic groin pain after groin hernia surgery ranges between 10% and 15%, and women are more prone to this than men.^35,36^ In the present study, severe chronic pain was reported by 1 participant. Musculoskeletal pain may mimic chronic groin pain, and a thorough history and examination are recommended to differentiate the 2 types of pain.

### Strengths and Limitations

This study had several strengths. The internal validity was high because surgical procedures were performed by the same surgeons, and follow-up was done by the same blinded observer. Additionally, the follow-up rate of 100% after 2 weeks and 1 year minimized selection bias. Recruiting participants from the general population and carrying out the study in a public health care sector resulted in high external validity. The controlled circumstances of the study and a highly specialized team of hernia surgeons performing a very high volume of surgeries during a short period are limitations to the external validity. However, it is possible that if surgeons are trained, they can achieve similar results in other parts of Uganda and in other low-income settings.

The main study limitation was discontinuation of the study after inclusion and follow-up of the first 200 participants, thereby decreasing the power of the trial and limiting any comparisons between the interventions. In addition, most recurrences were in the first 82 participants, suggesting that there was a learning curve.

## Conclusions

Results of this RCT demonstrate that MOAM repair under local anesthesia as outpatient surgery was safe and effective in the study setting. The technique could be used in the absence of laparoscopy and merits further investigation. Intraoperative visualization of the femoral canal for femoral hernias in women is highly recommended.

## Notes

Supplement 1.
Trial Protocol.

Supplement 2.
Statistical Analysis Plan.

Supplement 3. **eFigure.** Consort Flow Chart Per-Protocol Analysis **eTable 1.** Intraoperative Groin Hernia Findings Among Study Participants Stratified by Allocation Arm and by Intention-to-Treat and Per-Protocol Analysis **eTable 2.** Recurrent Hernias in 11 Study Participants—Index Operation and Findings at Reoperation **eAppendix.** Participants Who Died Before the 1-Year Follow-Up **eTable 3.** Hernia Recurrence and Death Stratified According to the Allocation Arm, Per- Protocol Analysis

Supplement 4.
Data Sharing Statement.
