# Supplementary material for: Open Anterior Mesh Repair vs Modified Open Anterior Mesh Repair for Groin Hernia in Women: A Randomized Clinical Trial
Source: JAMA Surg. 2025 Jul 16;160(9):946–53. doi: 10.1001/jamasurg.2025.2244 (PMC12268526; doi:10.1001/jamasurg.2025.2244)
Supplement: Supplement 1. — Trial Protocol. [file jamasurg-e252244-s001.pdf]

ORIGINAL PROTOCOL.

Open anterior mesh repair versus modified open anterior mesh repair for groin hernia in women. A double blinded randomized controlled trial.

2019

Title page

Title of study: Open anterior mesh repair versus modified open anterior mesh repair for groin hernia in women. A double blinded randomized controlled trial.

Investigators:

1. Alphonsus Matovu
   Mubende Regional Referral Hospital
   Plot M.4 Kakumiro Road
   P.O Box 4, Mubende, Uganda
   E-mail: alphonsusing@gmail.com
   Phone: +256 774 287 185

2. Jenny Löfgren, MD PhD
   Department of Molecular Medicine and Surgery
   Karolinska University Hospital, Solna (L1:00)
   SE-171 76 STOCKHOLM   901 85 Umeå, Sweden
   E-mail: jenny.loefgren@gmail.com
   Phone: +46-704 612426

3. Andreas Wladis, MD, PhD
   Department of clinical and experimental medicine
   Linköping University
   58183 Linköping
   E-mail: awladis@gmail.com
   Phone: +46-700 017539

5. Pär Nordin, MD, PhD
   Department of surgery and perioperative sciences
   University hospital of Umeå, Building 10:1
   901 85 Umeå, Sweden
   E-mail: par.nordin@regionjh.se
   Phone: +46-706 933873

Institutions:

The work will be conducted as collaboration between Makerere University, Uganda and Umeå University, Linköping University and Karolinska Institute, Sweden.

TABLE OF CONTENTS

Title page i
Operational definitions vi
Abstract vii
Background vii
Methods vii
Objective of the study vii
Introduction 1
1. Background to the study 1
2. Statement of the problem 2
3. Justification of the study 2
4. Conceptual framework 3
Fig 1. Conceptual framework 3
4.1. Consort Flow Chart. 4
5. Research questions 5
6. Objectives of the study 5
6.1. General objective of the study 5
6.2. The specific objectives are to 5
6.3. Hypothesis 5
7. Literature review 5
8. Methodology 7
8.1. Study design 7
8.2. Allocation ratio to treatment 7
8.3. Surgical methods and materials 8
8.4. Description of intervention for each study arm 8
8.4a –The open anterior mesh repair 8
8.4b The modified open anterior mesh repair for a groin hernia 9
8.5. Postoperative management 9
8.6 Follow up 10
8.7. Study areas 10
8.8. Study population 10
8.8a. Inclusion criteria: 10

8.8b. Exclusion criteria 11
8.9. Sample size and calculation 11
8.10. Sampling procedures 11
8.11. Randomization method 12
8.13. Assessment of safety 13
8.14. Adverse events reporting 13
8.15. Data safety and monitoring plan 13
8.16. Quality assurance 13
8.17. Funding source 13
9. Study variables 14
9.1. Primary endpoints: 14
9.2. Secondary endpoints: 14
10. Data collection 15
10.1. Figure 3. Timeline for study activities involving patients. 16
11. Recruitment 16
11.1. Participant recruitment 16
11.2 Training of research assistants 16
11.3 Tools 16
11.4 Pre-testing 16
11. 5 Field editing of data 17
11.6 Missing data 17
11. 7 Data management and analysis 17
11.8 Data entry and cleaning 17
11.9 Analysis plan and dissemination plan 17
12. Ethical considerations 18
References 20
APPENDICES 24
Appendix 1. Consent form(for participant) 24
Appendix 2: Participant contact information (for participant). 28
Appendix 3: The Medical History Form (for participant) 30
Appendix 4: Preoperative Physical Examination(for participant) 35
Appendix 5: Intervention protocol (for participant) 38
Appendix 6: Operation description for mesh repair method(for participant) 41

Appendix 7. Discharge form(for participant) 44
Appendix 8. Follow up 2 weeks(for participant)..45
Appendix 9. Follow up 1 year..49
Appendix 10. Follow up after 3 years..56
Appendix 11. Post operative information for patients 64
Appendix 12:WHO Surgical safety check list. 66
Appendix 13: Adverse event classification and reporting tool. 67
Appendix 14: Costing of groin hernia repair methods..70
Appendix 15: Work plan for the study. 72
Addendum 1: Endagano yo kwikiriza mu Lusoga. Lusoga consent form..............................73
Addendum 2 :Acholi translation. 78
Addendum 3: Luganda translation. 95
Addendum 4: Lugbara translation 115

Acronyms and Abbreviations

I/M HDSS	Iganga / Mayuge Districts Health and Demographic Surveillance Site
RRH	Regional Referral Hospital
GH	General Hospital
USD	United States Dollar
SSA	Sub-Saharan Africa
ASA	American Society of Anaesthesiologists

Operational definitions

Groin hernia	Inguinal and/or femoral hernia

Abstract

Background

Surgery has been considered too costly and with little impact on the burden of disease in resource limited settings. Research is refuting this belief and it has been found that surgical services are highly cost-effective and compare favourably with other prioritized areas in health care in such settings. Globally, 220 million people live with groin hernia. Around 20 million of these are women. Methods used for groin hernia repair in men, like the modified Bassini, the Lichtenstein and the Lockwood methods are not optimal in women who face elevated risks of recurrence following surgery compared to men.

Methods

In this double blinded randomised controlled trial, 440 women will be randomised into the control group (n=220) for the open anterior mesh repair or to the intervention group (n=220) for the modified open anterior mesh repair. The trial will be carried out in different hospitals in the country. Randomisation will be computer based. Primary endpoints are chronic pain and hernia recurrence. Secondary endpoints are patient satisfaction, costs and cost-effectiveness. A blinded observer will follow up the participants two weeks, one year, and three years' post operatively.

Objective of the study

This study is undertaken to develop an improved method for and open groin hernia repair in women in Uganda and other resource constrained countries by comparing the open anterior mesh repair against the modified anterior mesh repair for groin hernias in Women. Very little evidence on hernia repair in women exists despite that this is a large patient group. The findings will be used to guide the writing of clinical guidelines as well as training of surgical providers, primarily in sub-Saharan Africa.

Introduction

1. Background to the study

Global surgery is a field of research, advocacy and policy making (Bickler and Spiegel, 2008). Five billion people lack access to safe surgery at an affordable cost when needed yet surgery contributes to one third of the global burden of disease (Meara *et al.*, 2015). Surgery has been believed to be too costly for low income settings but this is a misunderstanding as surgery is as cost-effective as other prioritised health care interventions (Bae, Groen and Kushner, 2011)(Debas *et al.*, 2006).

Only a fraction of the world's surgeries occur in low-income settings (Weiser *et al.*, 2008). This results in an accumulation of disease that could have been amenable by surgery. Many deaths could also be averted by surgery (Groen *et al.*, 2012). The associated loss in productivity will amount to over 20 trillion USD between 2015 and 2030. Expressed in proportion of GDP Low- and Middle Income Countries (LMICs) are most affected (Alkire *et al.*, 2015).

In this context, conditions that are common and that can be treated at low cost, with high cost-effectiveness even in smaller health facilities should be prioritised. Groin hernia is such a condition.

Over 200 million people live with a groin hernia worldwide and if left untreated, this condition causes considerable pain and also leads to 40,000 deaths per year (Beard *et al.*, 2015). Epidemiological studies among women have not been performed to determine the real burden of disease but a previous study in Eastern Uganda indicated groin hernias in women contributing 24% of groin hernia surgery volume (Löfgren *et al.*, 2014). A facility based study carried out in 29 hospitals in Uganda found that 16% of the groin hernia repairs were performed in women, mainly using tension techniques (manuscript). Tension groin hernia repairs have high rates of recurrence compared to mesh repair (M P Simons *et al.*, 2009).

Even for mesh hernia repair, the recurrence rate is higher in women than in men (Nilsson, Holmberg and Nordin, 2017). A possible explanation is that it is a result of femoral hernias being relatively more common in women than in men, and that these may be overlooked during hernia repair. In high resource settings, laparoscopic repair with mesh implantation

has been found to carry the lowest rates of recurrence in women and is therefore the recommended approach in women (Nilsson, Holmberg and Nordin, 2017)(The HerniaSurge Group, 2018). Through this method, both the inguinal and femoral regions can be inspected and hernias repaired. However, due to high costs of equipment and maintenance as well as long learning curves compared to open techniques, laparoscopic hernia repair will not be available to the majority of the patients in Sub-Saharan Africa and Uganda for a long time to come. Developing and evaluating an open method, designed for women and can be utilized in Uganda and other Sub Saharan countries is therefore an urgent priority.

2. Statement of the problem

Groin hernia repair is the commonest general surgical procedure performed globally (Beard *et al.*, 2015). It affects children, women and men but most research has been carried out in men. The risk of recurrence is higher in women than in men when the same methods are used (Nilsson, Holmberg and Nordin, 2017). In the emergency setting, mortality is higher for women than for men undergoing hernia repair (Nilsson, Stylianidis, Haapamäki, *et al.*, 2007). This inequity warrants further investigation and correction.

Laparoscopic approach is considered golden standard in mesh hernia repair in women (M. P. Simons *et al.*, 2009). This method is not available to the majority of the patients in sub-Saharan Africa or Uganda. An open method for repair of groin hernia in women, which is easy to learn, safe to use and with high cost-effectiveness is therefore called for.

The proposed study will investigate the use of a modified anterior mesh technique that will cater for both inguinal and femoral hernias, hoping that we can reduce recurrence and chronic groin pain after groin hernia repair in women.

3. Justification of the study

This study aims to improve outcomes after groin hernia repair in women. The findings will be used to promote a new method for groin hernia repair in women in Low Income Settings. Promotion of the method will also promote groin hernia surgery in women subsequently reducing morbidity and mortality associated with groin hernias.

4. Conceptual framework

Fig 1. Conceptual framework

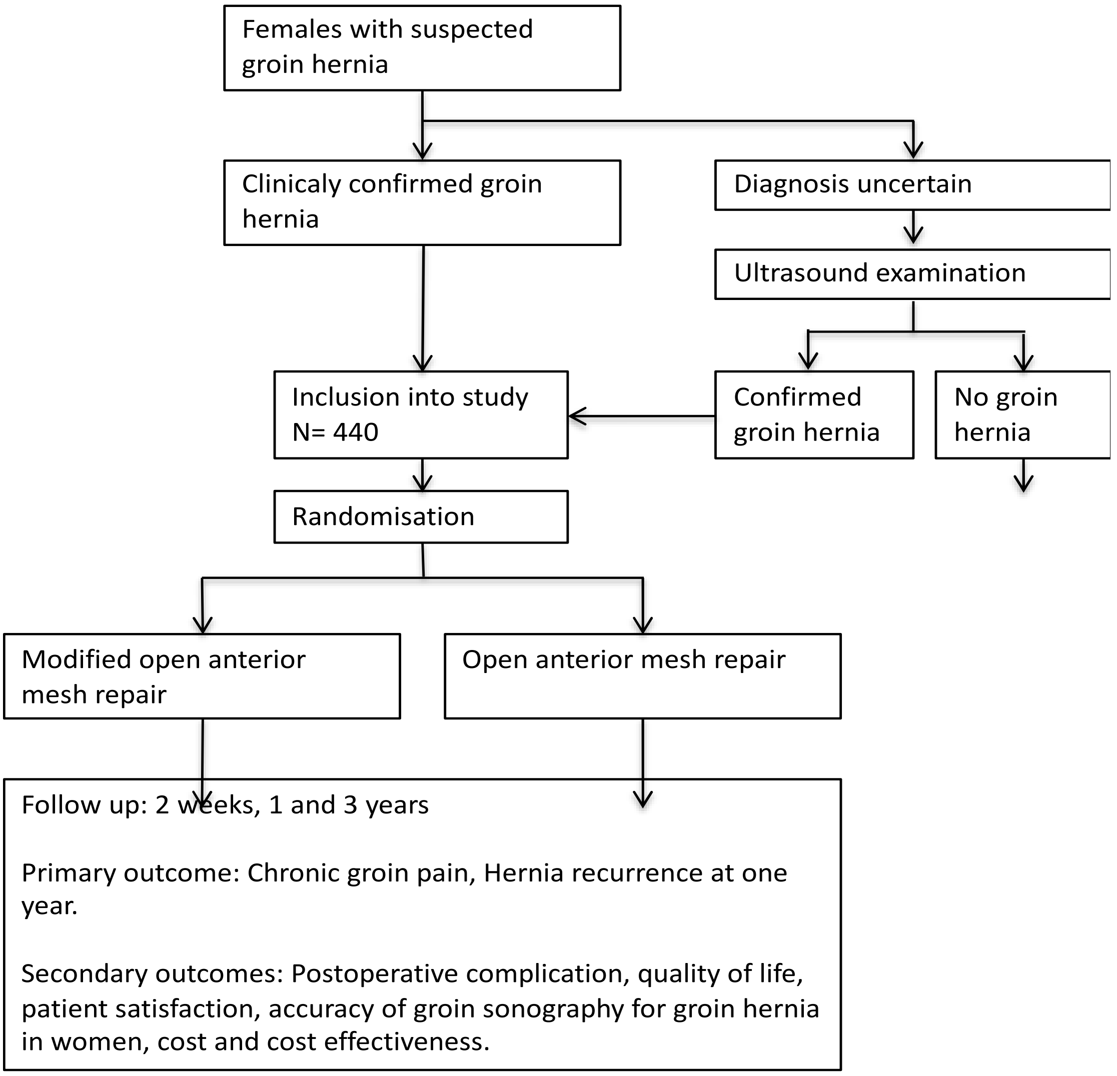

Compared to the original protocol, only uncertain cases will have an ultrasound examination.

4.1. Consort Flow Chart.

Fig. 2. Flow diagram of the progress through the phases of the study.

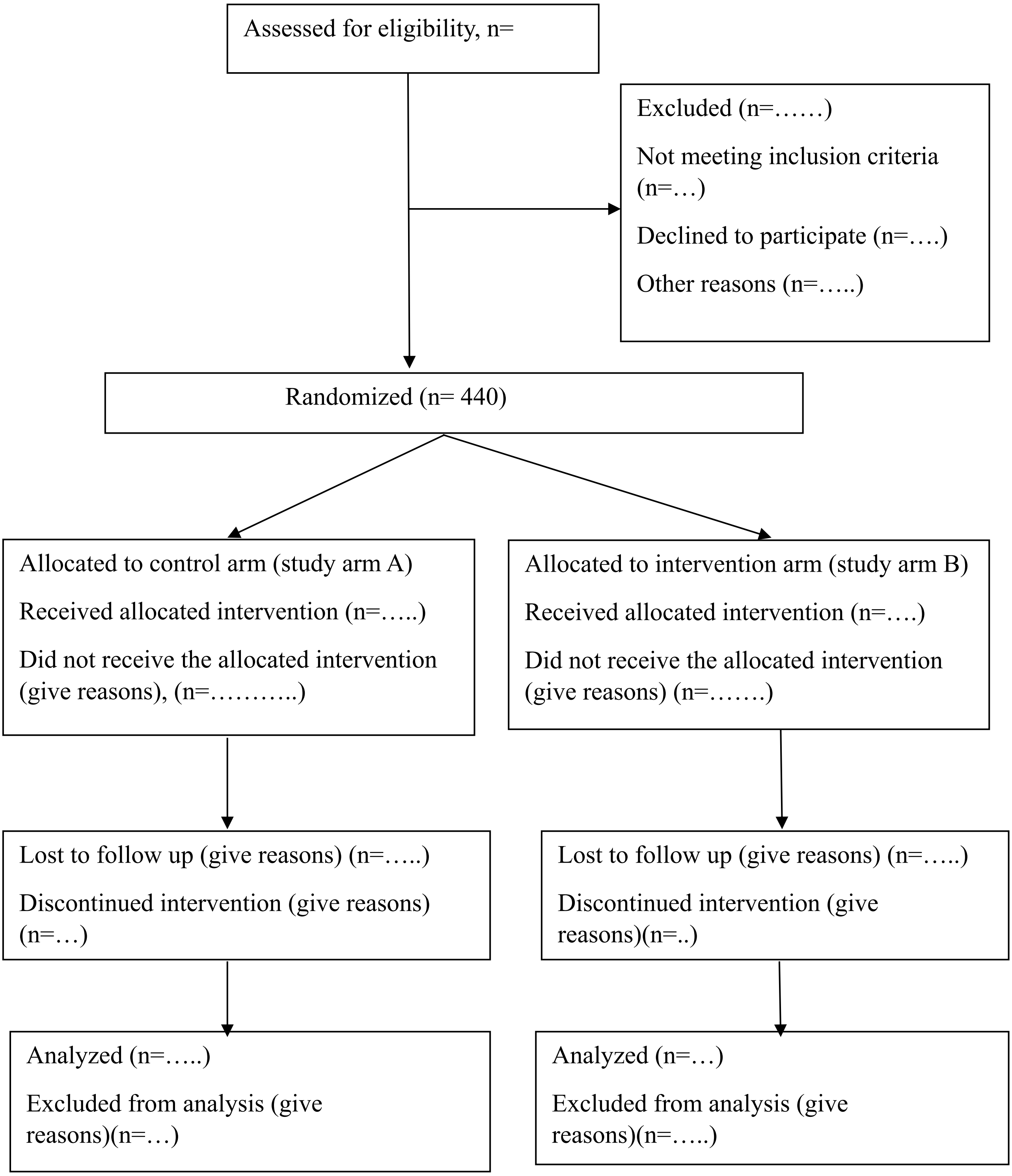

5. Research questions

i. What is the difference in safety and effectiveness of the open anterior mesh repair compared to the modified open anterior mesh repair for groin hernias in women?

ii. What is the difference in cost and cost effectiveness of the open anterior mesh repair compared to the modified open anterior mesh repair for groin hernias in women?

6. Objectives of the study

6.1. General objective of the study

The general objective of this study is to contribute with scientific evidence best practices in groin hernia repair in women.

6.2. The specific objectives are to

a) Evaluate and compare the outcomes after groin hernia repair using the open anterior mesh technique and the modified anterior mesh technique for groin hernia repair in women.

b) Calculate and compare costs and cost-effectiveness of this procedure (of the open anterior mesh technique with the modified anterior mesh technique for groin hernia repairs in women.

6.3. Hypothesis

There is no difference between the open anterior mesh repair and the modified open anterior mesh repair for groin hernias in women.

7. Literature review

Groin hernias in women can present as inguinal, femoral or inguinal femoral. Some of the factors associated with inguinal hernias in women in the United States of America are older age, rural residence, greater height, chronic cough and presence of an umbilical hernia(Ruhl and Everhart, 2007). These factors have not been studied in Uganda but groin hernias commonly present as groin swellings and they are also responsible for some pelvic pain in women(Perry and Echeverri, 2006). Persistent groin pain in women without a palpable lump should arouse suspicion of an occult hernia(Kark and Kurzer, 2008). Women have a high morbidity and mortality following groin hernias(Nilsson, Stylianidis, Haapamäki, *et al.*, 2007) but also women have a higher risk of recurrence (inguinal or femoral than men)

following an inguinal hernia operation due to a higher occurrence of femoral hernias(Nilsson, Holmberg and Nordin, 2017). It is therefore recommended that in female patients, the existence of a femoral hernia should be excluded in all cases of a hernia in the groin(M. P. Simons *et al.*, 2009).

Each year, over 20 million hernia repairs are undertaken worldwide and it is the world's most commonly performed general surgical procedure(The HerniaSurge Group, 2018). In high income settings, the lifetime risk of needing a groin hernia repair is 27% in men and 3% in women(PRIMATESTA and GOLDACRE, 2018). We have shown, as have others, that the prevalence of groin hernia in men is around 10% (Löfgren et al, 2014, Ohene-Yeboah et al, Abramson *et al.*, 1978). The prevalence of groin hernia in women has never been verified by epidemiological surveys including clinical examination(PRIMATESTA and GOLDACRE, 2018) (Patel *et al.*, 2014).

In high income settings women account for less than 10% of all adult groin hernia patients (Nilsson *et al.*, 2011). In some low income settings, however, women constitute up to 40% of patients undergoing hernia surgery (Löfgren *et al.*, 2014). Therefore, it is reasonable to assume that 5-10% groin hernia patients worldwide are women. This translates into 10-20 million women living with groin hernia and 1-2 million being operated for this condition each year. In addition, women with groin hernias are more often operated on as emergencies than men and face higher risks of adverse events including death (Nilsson *et al.*, 2011)(Nilsson, Stylianidis, Haapamäki, *et al.*, 2007). In resource constrained countries, mortality associated with emergency repair of strangulated hernia can be as high as 40% (Mbah, 2007). Watchful waiting is not recommended in women due to the higher risk of incarceration and strangulation (M P Simons *et al.*, 2009).

It is well established that anterior mesh repair according to Lichtenstein is the open method of choice in men with groin hernia (Scott *et al.*, 2002). In women on the other hand, this surgical approach is a risk factor for recurrence (Burcharth *et al.*, 2014). In Sweden, over 40% of women re-operated after primary inguinal hernia repair were found to have femoral hernias (Koch *et al.*, 2005), and femoral hernias occur earlier meaning that they are overlooked at the primary operation. In high income settings, a pre-peritoneal endoscopic or open approach is recommended in women as both inguinal and femoral hernias can be repaired that way (M P Simons *et al.*, 2009). Endoscopic hernia repair is more costly and resource demanding than open hernia repair. Therefore, it will not be available to most patients in low and middle

income countries within a foreseeable future and therefore it cannot be recommended in these settings. Open pre-peritoneal technique with mesh is difficult to master and therefore results are very dependent on the individual surgeon posing a substantial risk of recurrence (Koch *et al.,* 2005).

In Uganda, sutured techniques are the commonly used methods for groin hernia repair both in men and in women (manuscript). In women, a femoral hernia should be always excluded by laparoscopy or by open exploration of the pre- peritoneal space(Burcharth *et al.,* 2015).This ensures that femoral hernias are not overlooked. The suture methods used in inguinal hernia repair like the Shouldice and the Modified Bassini method do not recommend groin exploration for femoral hernias(Sachs, Damm and Encke, 1997). If a surgeon is operating on an inguinal or a femoral hernia that will be the focus of the operation. The infra inguinal approach has minimal access to the femoral canal allowing for recurrence of groin hernias in women. A safe and effective open technique which allows for exploration of both the inguinal and the femoral canal and that can be mastered by medical officers even in rural settings, is desired. This would have the potential to improve outcomes after groin hernia surgery in women in Uganda and other settings with limited financial and human resources.

8. Methodology

8.1. Study design

This is a parallel two arm double blinded randomized controlled trial. The control arm that will be treated with the open anterior mesh method and the intervention group will be treated with the modified open anterior mesh method for a groin hernia. The study is double blinded because the principal investigator will not be aware of the study arm of each of the participants and he will not take part in the surgical operations. The study participants will not know which method has been used to treat the groin hernia.

8.2. Allocation ratio to treatment

The participants will be randomized into two arms, the control arm and the intervention arm. The allocation ratio will be 1:1, or about 220 participants per study arm.

8.3. Surgical methods and materials

The aim is to perform all procedures under local anaesthesia and as day case surgeries. Conversion to general or spinal anaesthesia may be required if there is excessive patient discomfort or pain.

Prior to starting of the study, the participating surgeons will operate an estimated 50 cases together to ensure that all surgeons are operating using the exact same technique. The planned number of surgeons is 10 (5 Ugandan and 5 International). A low-weight, commercial mesh made of polypropylene will be used in all patients.

For all participants, skin preparation will be done using povidone iodine or 70% alcohol; local anaesthesia will be constituted by an equal mix of lidocaine (10mg per milliter) and ropivacaine (7.5mg per ml). A prophylactic oral antibiotic of 1.5gms of flucloxacillin will be administered one hour before surgery. For those who may have penicillin allergy, clindamycin 900 mgs will be administered one hour before surgery. One dose of 1 gram of paracetamol will be administered together with the antibiotics.

8.4. Description of intervention for each study arm

The two interventions for this study are a) the open anterior mesh repair (control group) and b) the modified open anterior mesh repair (the intervention group).

8.4a –The open anterior mesh repair

The open anterior mesh repair is done using the following approach. The inguinal canal is opened; the round ligament is identified and isolated. Do not excise the round ligament. The hernia sac is then identified and isolated. If an indirect sac is identified, it is opened to identify the contents. If contents are present, they are reduced; the sac is twisted until the turns reach the neck at the internal ring, this reduces the contents in the sac further into the peritoneal cavity. The sac is transfixed as proximal as possible and the stump is divided 1cm distal to the transfixing suture. A direct sac is inverted with a single absorbable invaginating suture.

Thereafter, the lower edge of the mesh is sutured by a continuous nylon 2.0 suture, which secures the mesh medially to the lacunar ligament and then proceeds laterally along the inguinal ligament beyond the internal ring. The superior edge of the mesh is loosely secured

to the internal oblique and the conjoint tendon with interrupted nylon 2.0 sutures. Laterally, the mesh is secured with interrupted nylon 2.0 to the inguinal ligament lateral to the inguinal ring, the internal oblique and the conjoint tendon. The external oblique will be repaired with a continuous absorbable Vicryl 0. Interrupted skin sutures will be used to repair the skin.

8.4b The modified open anterior mesh repair for a groin hernia

The modified open anterior mesh repair method is performed using the following approach. The inguinal canal is opened; the round ligament is identified and isolated. Do not excise the round ligament. The hernia sac is then identified and isolated. If an indirect sac is identified, it is opened to identify the contents. If contents are present, they are reduced; the sac is twisted until the turns reach the neck at the internal ring, this reduces the contents in the sac further into the peritoneal cavity. The sac is transfixed as proximal as possible and the stump is divided 1cm distal to the transfixing suture. A direct sac is inverted with a single absorbable invaginating suture. The medial aspect of the transversalis fascia is opened in order to allow for exploration of the femoral canal. A thin part of a low weight mesh is extended into the area to cover the femoral canal where a femoral hernia may arise. It is sutured to the ligament of cooper (pectineal ligament, using non absorbable suture nylon 2.0. The lower edge is sutured by a continuous nylon 2.0 suture, which secures the mesh medially to the lacunar ligament and then proceeds laterally along the inguinal ligament beyond the internal ring. The superior edge of the mesh is loosely secured to the internal oblique and the conjoint tendon with interrupted nylon 2.0 sutures. Laterally, the mesh is secured with continuous nylon 2.0 to the inguinal ligament lateral to the inguinal ring, the internal oblique and the conjoint tendon. The external oblique is repaired with continuous vicryl 0. Interrupted skin sutures will be used to repair the skin.

8.5. Postoperative management

Immediately after operation, the participants receive the next dose of oral analgesia (paracetamol and ibuprofen). All patients will receive oral paracetamol 1gm every 6hours for the next 5 days as well as ibuprofen 400 mg every 8 hours for 5 days. When the pain subsides, the frequency of using pain medication will reduce and most will not need to use analgesia for more than 5 days.

At least two hours after operation, a medical doctor will assess the patients to determine if they can be discharged from the hospital. Those unable to go home will be observed in the hospital until they are fit to leave. Patients operated late in the day will also stay overnight depending on travel distance to go home.

All patients will be instructed to keep the dressing for 7 days. On the 7th day, they will remove the dressing by themselves. Prior to discharge from the hospital, all patients will receive written and oral information about what to expect during the recovery period, warning signs and a scheduled date and time for follow up. The patients will be given a phone number to contact the study team if questions or complications arise.

8.6 Follow up

Follow up will take place for all patients after 14 days, 1 year and 3 years. The follow up will consist of interviews performed by the research assistants using the study tools. Thereafter, a physical examination will be performed by a physician. At the 14 days follow up, the sutures will also be removed. Patients will mostly be followed up at the point of inclusion into the study. If necessary, patients may also be seen from other sites including their homes.

8.7. Study areas

The study will be carried out in 11 hospitals in Uganda some of which are Regional Referral Hospitals and others are General Hospitals provided they have an operating theatre with the Uganda Ministry of Health Standards. The hospitals are Mubende Regional Referral Hospital, Hoima Regional Referral Hospital, Jinja Regional Referral Hospital, Iganga Hospital, Buluba Hospital, Kitovu Hospital, Tororo Hospital, Kitgum Hospital, Lacor Hospital, Arua Regional Referral Hospital and Kamuli Mission Hospital. These hospitals have been selected depending on convenience to the research team.

8.8. Study population

8.8a. Inclusion criteria:

Adult women 18 years and above,(this is the age of consent and below 15yrs of age the body is still growing making it inappropriate to use mesh repair) with primary, reducible groin hernia, ASA class 1 and 2(Sankar *et al.*, 2014) and with ability to give a written informed consent.

8.8b. Exclusion criteria

Recurrent hernia, incarcerated groin hernia requiring emergency operation, known coagulopathy (including medically induced, but not including daily usage of low dose aspirin), known pregnancy, obvious alcohol or substance abuse. Only obvious drug and alcohol abuse is an exclusion criterion. Signs can range between the individual being drunk, smelling of alcohol and being in poor general condition. For the latter, it can be expected that they will belong to ASA class 3 or higher which in itself is an exclusion criteria. Lab tests will not be used to diagnose drug or alcohol abuse.

8.9. Sample size and calculation

This trial has a superiority design. With 80% power, and 5% precision rate, and an expected success in the intervention arm of 99% after one year and 94% in the control arm, a sample size of 418 study participants is required. To compensate for un expected loss to follow up of 5%, 440 patients will be enrolled into the study. The calculation is based on the formula below. The calculation was done using the online tool "sealed envelope Ltd 2012".

$$n = f(\alpha/2, \beta) \times [p_1 \times (100 - p_1) + p_2 \times (100 - p_2)] / (p_2 - p_1)^2$$

$$f(\alpha, \beta) = [\Phi^{-1}(\alpha) + \Phi^{-1}(\beta)]^2$$

p1 and p2 are proportion of expected success rate in control and intervention group respectively, β=power, α=significance level, Φ^{-1} is the cumulative distribution function of a standardised normal deviate.

8.10. Sampling procedures

Mobilization will be done over radio and through raising awareness by the local leadership as well as through the study hospitals and other health facilities in the vicinity of the study hospitals. Potential study participants will present to the recruitment site, most commonly the study hospital. There they will be informed about the study and will get the chance to ask question in group and individually. The potential study participants will be assessed for eligibility to be included into the study. Those who fulfill the criteria or those where the

physical examination is inconclusive will receive a date for when they should present at the study hospital.

That day, the following will take place:

a. Informed consent
b. Interviews using study questionnaires
c. Physical examination to confirm that inclusion criteria are met
d. Ultrasonography of the groins will be done for all study participants. Individuals where the physical examination has been inconclusive will be offered ultrasonography before inclusion into the study as individuals who do not have a groin hernia are not eligible study participants.

Those who meet the eligibility criteria and who have given their consent to be part of the study will remain in the hospital for operation on the following day. A theatre list for the following day's operations will be generated. On the day of the operation, patients will be distributed consecutively to the surgeons according to the predetermined theatre list.

8.11. Randomization method

Randomization for the surgical method to be applied will be done after the surgeon and the patient he or she is going to operate on have entered the operating room. A computer based program will be used to randomize the sequence of treatment groups in blocks of four and six and eight. The allocation ratio between the study arms will be 1:1. After randomization, the surgeon will be informed which method to use. If a femoral hernia is detected intra operatively in a patient randomized to the control group (study arm A) the patients will cross over to the intervention group (study arm B). The physicians doing the follow up of the patients will not take part in randomization or the operations. The patients will not be informed which study arm they have been allocated to. Thus the patients and the physicians doing the follow up will be blinded to the treatment arm of the patient in this study.

8.12. Blinding mechanism

The physicians doing the follow up of the patients will not take part in randomization or the operations. The patients will not be informed which study arm they have been allocated to. Thus, the patients and the physicians doing the follow up will be blinded to the treatment arm of the patient in this study.

8.13. Assessment of safety

The WHO checklist for safe surgery (appendix 12) will be used in the operating room. The patients will be monitored in the hospital after the operation for 2 hours. This is a normal duration of postoperative monitoring for elective day case surgery. Major bleeding or complications with severe pain will be detected within that time interval. Only patients who are fit to go home will be discharged from the hospital. The patients will receive oral and written information about the time after the procedure, including expected recovery and warning signs. The patients will be given a phone number to the principal investigator which they can call if needed. If a complication has occurred, they will receive instructions on what to do. An extra assessment will be done if that is required.

8.14. Adverse events reporting

Adverse events will be investigated and reported to the Principal Investigator and Ms Harriet Chemusto chairperson MUREC. An adverse assessment and records tool will be used to record and report all adverse events among the study participants (appendix 13).

8.15. Data safety and monitoring plan

Each patient will be given a study number and the data will be kept in a locked box during field work, the key with the Principle Investigator. After data entry and scanning of forms containing only the study number, the forms will be delivered to Mildmay Uganda for storage for 10 years.

8.16. Quality assurance

The Principal Investigator is responsible for the collection and quality of the data. After each day, study forms will be reviewed and missing information will be retrieved. Correction of errors is the responsibility of the Principle Investigator.

8.17. Funding source

The study is funded through grants for development studies and awarded by the Swedish Research Council. The Swedish Research Council is Sweden’s largest governmental research funding body, and supports research of the highest quality within all scientific fields. For further

details, please see: https://www.vr.se/english. The same grant has also been used to fund hernia research in Ghana and in Sierra Leone. The grant was awarded to Prof Andreas Wladis, one of the co-investigators and is managed by Linköping University

9. Study variables

9.1. Primary endpoints:

Ppostoperative complications are excessive pain of scores 8,9 and 10 using the Visual Analogue Scale, chronic pain, hematoma/bleeding, infection, seroma formation, urinary retention, and others judged to be so at two weeks and hernia recurrence after one and three years. These end points will be used to determine the safety and effectiveness of both methods which will be compared between both arms of the study.

9.2. Secondary endpoints:

Patient satisfaction and quality of life at one and three years compared to before the surgery. Accuracy of groin sonography for female groin hernias will be determined. Cost and cost effectiveness will be calculated based on outcomes at one year follow up.

Cost-effectiveness will be expressed as cost in USD per DALY averted and QALY gained. These will be calculated as follows:

i. Costs of the interventions. This will include cost of medicines and materials, staff costs, capital costs and overhead costs (appendix 14)
ii. DALYs will be calculated using the following formula:

DALY = YLD + YLL
YLD = DW X remaining life expectancy at the time of surgery
YLL = risk of early death without surgery X remaining life expectancy at time of surgery

The results from the Inguinal Pain Questionnaire which is part of the interview tools will be translated into three levels of abdominopelvic problem (mild, moderate and severe) according to the global burden of disease study. The values for the most recent version of the Global Burden of Disease Study at the time of data analysis will be used. Remaining life expectancy at the time of the surgery will be retrieved from the WHO Life Table for Uganda. In the lack of information on the risk of incarceration of groin hernia in women, the same value as for men will be used but a sensitivity analysis will also be used, where the expected risk of death is modulated.

DALYs averted will be calculated using the difference of YLD and YLL between the preoperative assessment and the one year follow up, multiplied with the remaining life expectancy at the time of the operation.

iii. QALYs will be calculated using the following formula:

QALY = index value X remaining life expectancy at time of surgery

The results from the EQ5D form that is part of the interview tools will be translated into index values using the EQ5D Index Value Translator from the EuroQol group website. Remaining life expectancy at the time of the surgery will be retrieved from the WHO Life Table for Uganda. QALYs gained will thereafter be calculated using the difference in index value before and 1 year after surgery multiplied by the remaining life expectancy at the time of surgery

These methods were used in a previous study on groin hernia surgery in men in Uganda and we are also using it for cost effectiveness analysis in our trials in Ghana and Sierra Leone (Löfgren *et al.*, 2017). Using the same method will enable comparison between countries. We expect that the modified version of the anterior mesh repair will be superior to the original anterior mesh repair in terms of recurrence rate. As the modified version involves surgery close to sensitive structures, the risk of particularly hematoma could be increased. We expect both methods to be highly cost effective but that the modified version will be more cost effective as we expect that the recurrence rate in this group will be lower than in the control group.

10. Data collection

Data will be collected at different stages of the study by the responsible team. Pre –operative data will be collected by the Principle Investigator and research assistants, intra operative data will be collected by the surgeon operating on the patient. The immediate post-operative data and ongoing follow up data will be collected by the Principle Investigator and the research assistants. Timeline for study activities is presented in Figure 3 below.

10.1. Figure 3. Timeline for study activities involving patients.

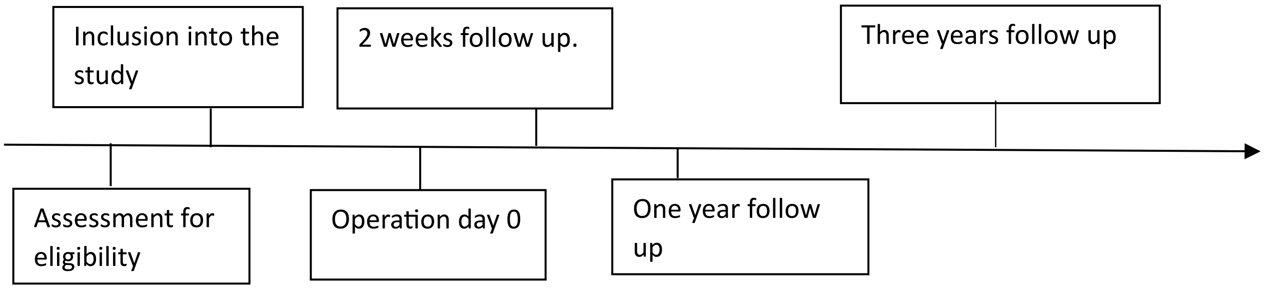

11. Recruitment

11.1. Participant recruitment

Participants will be mobilized using radios, village health teams and the local leaders. Radio stations will be given an approved announcement that will call upon women with groin swellings to come to the respective hospitals gazetted as a study site. All those that fulfill the inclusion criteria will fill a written consent form after a full explanation by the Principle investigator. The recruited participants will receive a general and specific examination by the Principal Investigator. Those with uncertain clinical examination findings will have groin sonography performed to verify or rule out groin hernia. Those with clinically or sonographically confirmed diagnosis will be randomized into the study.

11.2 Training of research assistants

Field assistants will be recruited to perform interviews with the patients at recruitment and follow up. The Principle Investigator is responsible for this training. The field assistants will not be working on their own but will be supervised by the Principle Investigator or another doctor involved in recruitment and follow up.

11.3 Tools

The tools consist of consent forms, contact information, questionnaires and forms for physical examination (See appendix 1 to 14). Answers have been coded, when possible.

11.4 Pre-testing

The tools were used previously in a double blinded randomised clinical trial of a low cost mesh in groin hernia repairs in men. They will be updated so that gender specific questions relate to women instead of men. Additional pre-testing is not necessary.

11. 5 Field editing of data

After each day, the questionnaires filled will be reviewed. This will enable to edit data for correctness and accuracy. Any inconsistent information will be discussed with the field assistants for correction.

11.6 Missing data

Any missing data will be discussed with the field assistants for filling and correction. Some information may have to be verified with a patient over the phone or at the next scheduled appointment.

11. 7 Data management and analysis

The data will be managed by observing strict collection protocols, entry, cleaning and analysis. The data will be stored securely in both hard and soft copies.

11.8 Data entry and cleaning

The Principal Investigator is responsible for the data entry and cleaning of the data. He will do large parts of it himself but may delegate this task to others in the research team, potentially including medical students. External staff for data entry will not be used. Data will be entered into excel spread sheets

11.9 Analysis plan and dissemination plan

Data analysis will be performed using primarily Excel and SPSS. Counts will be presented as numbers and per cent and comparison of binary values will be done using chi square test, Fischer exact test or an exact binomial test as appropriate. Continuous data will be presented as mean and standard deviation and analysis will be done using students t-test. Absolute difference between the study groups for the primary and secondary endpoints will be calculated and presented with 95% confidence intervals. A difference of more than 5 percentage points for the primary endpoint (recurrence) is considered clinically relevant. A p-value of 0.05 is considered statistically significant.

The PI is responsible for the quality control. The findings of the study will be disseminated through presentations in local and international conferences as well as publications in peer reviewed journals. The dissemination strategy also involves ministries of health, international organisations, policy makers and funders of global health initiatives. Information about the results to the study participants will be delivered at the time of the follow up (1 year and 3

years). A report will be made available to study participants and others through the study hospitals.

12. Ethical considerations

Ethical clearance has been obtained from the Mildmay Uganda Research and Ethics Committee accredited by the Uganda National Council of Science and Technology. Letters of administrative clearance will be obtained from the respective hospitals where the operations will be conducted before submission to Uganda National Council of Science and Technology.

Patients will receive oral and written information about the study in their local language. They will be included into the study after having given written consent. In case they cannot write, they will thumb print the consent form.

Confidentiality is assured for the study participants. Questionnaires will be stored at the Mildmay Uganda Research office. De-identification of data will be done at data entry. No one part from the research team and field assistants will be involved in the management of the data.

The main benefits to the study participants are receiving a hernia repair at no cost, close follow up and attending to any complications at no cost to the patient. There are always risks associated with surgery but these are not elevated by the present study. The potential risks expected include postoperative complications that are normally seen after groin hernia surgery. These include post operative bleeding with hematoma formation, seroma formation and wound infection. Long term complications include chronic pain and hernia recurrence. Sterility practices and the WHO surgical safety checklist will be used to reduce risks associated with surgery. The WHO surgical safety checklist is attached as appendix 12. At discharge, the patients will receive oral and written information about the post operative period. This includes what to expect and warning signs for when to contact the study team. They will be given a contact number of a doctor at the hospital where they had the operation and the principal investigator to call. If the patient needs to travel to hospital but cannot reach the hospital in a short time we shall facilitate her to travel. An additional visit to or by the study team may be carried out. Need for reoperation or surgical interventions for complications are very rare. Most complications will be handled conservatively with antibiotics or analgesics. In situations of hernia recurrence, the participant will be offered a re-operation by a surgeon experienced in this, at no cost to the patient.

References

Abramson, J. H. *et al.* (1978) 'The epidemiology of inguinal hernia. A survey in western Jerusalem', *Journal of Epidemiology and Community Health*, 32(1), pp. 59–67.

Alkire, B. C. *et al.* (2015) 'Global economic consequences of selected surgical diseases: a modelling study', *The Lancet. Global Health*, 3 Suppl 2, pp. S21-27. doi: 10.1016/S2214-109X(15)70088-4.

Bae, J. Y., Groen, R. S. and Kushner, A. L. (2011) 'Surgery as a public health intervention: common misconceptions versus the truth', *Bulletin of the World Health Organization*, 89(6), p. 394. doi: 10.2471/BLT.11.088229.

Beard, J. H. *et al.* (2015) 'Hernia and Hydrocele', in Debas, H. T. et al. (eds) *Essential Surgery: Disease Control Priorities, Third Edition (Volume 1)*. Washington (DC): The International Bank for Reconstruction and Development / The World Bank. Available at: http://www.ncbi.nlm.nih.gov/books/NBK333501/ (Accessed: 29 April 2018).

Bickler, S. W. and Spiegel, D. A. (2008) 'Global surgery—defining a research agenda', *The Lancet*, 372(9633), pp. 90–92. doi: 10.1016/S0140-6736(08)60924-1.

Burcharth, J. *et al.* (2014) 'Direct inguinal hernias and anterior surgical approach are risk factors for female inguinal hernia recurrences', *Langenbeck's Archives of Surgery*, 399(1), pp. 71–76. doi: 10.1007/s00423-013-1124-z.

Burcharth, J. *et al.* (2015) 'Patient-Related Risk Factors for Recurrence After Inguinal Hernia Repair: A Systematic Review and Meta-Analysis of Observational Studies', *Surgical Innovation*, 22(3), pp. 303–317. doi: 10.1177/1553350614552731.

Debas, H. T. *et al.* (2006) ‘Surgery’, in Jamison, D. T. et al. (eds) *Disease Control Priorities in Developing Countries*. 2nd edn. Washington (DC): World Bank. Available at: http://www.ncbi.nlm.nih.gov/books/NBK11719/ (Accessed: 27 October 2013).

Groen, R. S. *et al.* (2012) ‘Untreated surgical conditions in Sierra Leone: a cluster randomised, cross-sectional, countrywide survey’, *Lancet*, 380(9847), pp. 1082–1087. doi: 10.1016/S0140-6736(12)61081-2.

Kark, A. E. and Kurzer, M. (2008) ‘Groin hernias in women’, *Hernia*, 12(3), pp. 267–270. doi: 10.1007/s10029-007-0330-4.

Koch, A. *et al.* (2005) ‘Prospective evaluation of 6895 groin hernia repairs in women’, *British Journal of Surgery*, 92(12), pp. 1553–1558. doi: 10.1002/bjs.5156.

Löfgren, J. *et al.* (2014) ‘Prevalence of treated and untreated groin hernia in eastern Uganda’, *The British Journal of Surgery*, 101(6), pp. 728–734. doi: 10.1002/bjs.9457.

Löfgren, J. *et al.* (2017) ‘Cost-effectiveness of groin hernia repair from a randomized clinical trial comparing commercial versus low-cost mesh in a low-income country’, *The British Journal of Surgery*. doi: 10.1002/bjs.10483. Manuscript. Groin hernia surgery in Uganda. A hospital based survey on the volumes and practices.

Mbah, N. (2007) ‘Morbidity and mortality associated with inguinal hernia in Northwestern Nigeria’, *West African Journal of Medicine*, 26(4), pp. 288–292.

Meara, J. G. *et al.* (2015) ‘Global Surgery 2030: evidence and solutions for achieving health, welfare, and economic development’, *Lancet*. doi: 10.1016/S0140-6736(15)60160-X.

Nilsson, H., Stylianidis, G., Haapam??ki, M., *et al.* (2007) 'Mortality After Groin Hernia Surgery':, *Annals of Surgery*, 245(4), pp. 656–660. doi: 10.1097/01.sla.0000251364.32698.4b.

Nilsson, H., Stylianidis, G., Haapamäki, M., *et al.* (2007) 'Mortality after groin hernia surgery', *Annals of Surgery*, 245(4), pp. 656–660. doi: 10.1097/01.sla.0000251364.32698.4b.

Nilsson, H. *et al.* (2011) 'Mortality after groin hernia surgery: delay of treatment and cause of death', *Hernia: The Journal of Hernias and Abdominal Wall Surgery*, 15(3), pp. 301–307. doi: 10.1007/s10029-011-0782-4.

Nilsson, H., Holmberg, H. and Nordin, P. (2017) 'Groin hernia repair in women – A nationwide register study', *The American Journal of Surgery*. doi: 10.1016/j.amjsurg.2017.07.027.

Ohene-Yeboah, M. *et al.* (2016) 'Prevalence of Inguinal Hernia in Adult Men in the Ashanti Region of Ghana', *World Journal of Surgery*, 40(4), pp. 806–812. doi: 10.1007/s00268-015-3335-7.

Patel, H. D. *et al.* (2014) 'An estimate of hernia prevalence in Sierra Leone from a nationwide community survey', *Hernia: The Journal of Hernias and Abdominal Wall Surgery*, 18(2), pp. 297–303. doi: 10.1007/s10029-013-1179-3.

Perry, C. P. and Echeverri, J. D. V. (2006) 'Hernias as a Cause of Chronic Pelvic Pain in Women', p. 4.

PRIMATESTA, P. and GOLDACRE, M. J. (2018) 'Inguinal Hernia Repair: Incidence of Elective and Emergency Surgery, Readmission and Mortality', *INTERNATIONAL JOURNAL OF EPIDEMIOLOGY*, p. 5.

Ruhl, C. E. and Everhart, J. E. (2007) ‘Risk Factors for Inguinal Hernia among Adults in the US Population’, *American Journal of Epidemiology,* 165(10), pp. 1154–1161. doi: 10.1093/aje/kwm011.

Sachs, M., Damm, M. and Encke, A. (1997) ‘Historical evolution of inguinal hernia repair’, *World Journal of Surgery,* 21(2), pp. 218–223. doi: 10.1007/s002689900220.

Sankar, A. *et al.* (2014) ‘Reliability of the American Society of Anesthesiologists physical status scale in clinical practice’, *British Journal of Anaesthesia,* 113(3), pp. 424–432. doi: 10.1093/bja/aeu100.

Scott, N. W. *et al.* (2002) ‘Open mesh versus non-mesh for repair of femoral and inguinal hernia’, *The Cochrane Database of Systematic Reviews,* (4), p. CD002197. doi: 10.1002/14651858.CD002197.

Simons, M P *et al.* (2009) ‘European Hernia Society guidelines on the treatment of inguinal hernia in adult patients’, *Hernia: the journal of hernias and abdominal wall surgery,* 13(4), pp. 343–403. doi: 10.1007/s10029-009-0529-7.

Simons, M. P. *et al.* (2009) ‘European Hernia Society guidelines on the treatment of inguinal hernia in adult patients’, *Hernia*, 13(4), pp. 343–403. doi: 10.1007/s10029-009-0529-7.

The HerniaSurge Group (2018) ‘International guidelines for groin hernia management’, *Hernia*, 22(1), pp. 1–165. doi: 10.1007/s10029-017-1668-x.

Weiser, T. G. *et al.* (2008) ‘An estimation of the global volume of surgery: a modelling strategy based on available data’, *Lancet*, 372(9633), pp. 139–144. doi: 10.1016/S0140-6736(08)60878-8.

APPENDICES

Patient study number

Appendix 1. Consent form(for participant)

Introduction: My name is ………………………., and I am part of the team conducting a study entitled open anterior mesh repair versus modified open anterior mesh repair for groin hernia in women. A double blinded randomized controlled trial. This study has been approved by an accredited research and ethics committee (Mildmay Uganda Research and Ethics Committee)**.**

Purpose: Inguinal hernia is a common condition worldwide. Among men, the prevalence was almost 10% in a previous study. The surgery method that is commonly used to treat inguinal hernia in men uses a mesh to reinforce the abdominal wall and reduce the risk of recurrence. Very few studies evaluating the best surgical technique in women have been done but it is clear that the same method normally used in men is not optimal in women. In a previous study that we conducted on hernia surgery in men, we found that a low cost mesh was safe and effective. In this study we will evaluate if this mesh can be used also in women using a technique modified for female patients. We will compare this method with current mesh techniques that involve mesh.

Research procedure: We will recruit 440 women with groin hernia from the catchment populations of Mubende Regional Referral Hospital, Iganga hospital Hoima Regional Referral Hospital, Jinja Regional Referral Hospital, Buluba Hospital, Kitovu Hospital, Tororo Hospital, Kitgum Hospital, Lacor Hospital, Arua Regional Referral Hospital and Kamuli Mission Hospital. Half of the patients will be operated using the current open anterior mesh technique and the other half will be operated using the modified open anterior mesh

technique which is under investigation. The patients will not know which method was used. The surgeries will be performed by surgeons under local anaesthesia. This means that the patient will be awake during the surgery but will not feel pain. The operation will take between 60 to 120 minutes. After the surgery, the patient is able to move freely and it is safe to go home the same day. After 2 weeks, after one year and after 3 years, the patient will be interviewed and thereafter examined by a medical doctor to assess the results of the surgery. The participants will get a feedback on the progress and findings of the study

Potential benefits from this study: You will receive surgery for your hernia free of charge. You will receive UGX 20,000 to facilitate your transport on every visit to the study site to participate in the study, i.e., pre –enrolment, two weeks post enrolment when you turn up for review, one year post enrolment for the one year review and 3 years post enrolment for the 3 year review. If any complications occur, and you cannot reach the hospital in a short time, we will facilitate you to travel, and for all complications we will provide you with correct and effective treatment. You will receive follow up after 2 weeks and one year so that potential complications can be found and treated in time.

Potential harm from this study: All surgical interventions have some risks. Some patients have pain after the surgery and a small number will have chronic pain. Some may have bleeding from the wound or a haematoma, There is a risk of infection with pus discharge at the operation site. In some patients the hernia may recur. In case any complications occur, inform the Principle Investigator and travel back immediately to the study site for assistance. The risk for these complications will be reduced to a minimum through safety measures taken by the study team. A few patients may have recurrence of the hernia. We will provide a reoperation if that occurs.

Voluntary participation: Participation in this study is voluntary. If you choose to take part, I will first ask you to sign or finger print this document to verify your consent to participate. Thereafter, I will ask some questions about your health in general and your inguinal hernia in particular. Together, we will decide a date when you will come to the hospital nearest to you for an operation. The procedure will be performed the day after. A doctor will meet you for a medical control 2 weeks after the surgery, one year after the surgery and three years after surgery.

Confidentiality and autonomy: The information that you give us will be kept safely so that no one apart from the investigators have access to it. All data will be de-identified so that your name will not appear in the analysis or any resulting publications. If you have any questions about the study, please ask me. Also (PIs name and phone number) can be contacted for further questions. It is your decision to participate in the study and you are free to stop taking part in the study at any time without any consequences or penalty.

Questions regarding the rights of participants or any complaints about the research can be directed to Ms Harriet Chemusto phone number 0392-174-236, the chairperson of Mildmay Uganda Research and Ethics Committee.

Acceptance: Before we proceed with the interview, I would like to seek your permission. Do you consent to participate in the study?

I have been fully explained about this study and understand its purpose and objectives. I understand the details and have been informed about the requirements of the study. My questions have been answered satisfactory. I hereby agree to participate in the study.

Name of participant ……………………………………………….

Signature of participant ___________________________________

Date________________

Thumb print of respondent ---

Witness's signature if participant is illiterate

………………………………………..

Name of person obtaining the consent ………………………………………

Signature of person obtaining the consent _______________________________

Date________________

Appendix 2: Participant contact information (for participant).

Patient study number

Filled in by ____

Date and place ____

A. Identification of the patient

1.Name:____

2.Name of district____

3. Name of county……………………………………………………………………...

4. Name of subcounty………………………………………………………………….

5. Name of parish…………………………………………………………………………

6.Village name____

7.Mobile phone number 1____

8. Mobile phone number 2 ____

9. Mobile phone number 3 ____

10. Name of Next of kin……………………………………………………………….

11.Relationship. ………………………………………………………………………….

12.Phone number of next of kin……………………………………………………

13.Local chairman____

14. Phone number of local chairman____

15.Occupation____

16. Place of work (if any) ____

Patient study number

17.Additional contact information

____

____

____

Appendix 3: The Medical History Form (for participant)

Patient study number

Filled in by ____

Date and place ____

A. Identification of the patient

1.Name:____

2.Name of district____

3.Villagename____

4.Mobile phone number 1____

5. Mobile phone number 2 ____

6. Mobile phone number 3 ____

7.Local chairman____

8. Phone number of local chairman____

9.Occupation____

10. Place of work (if any) ____

11. Additional contact information

____

____

____

12.Emyaka Age in years ☐

13. Ofuwa sigara

Smoker 1=Yes, 2=No, 3=Stopped ☐

Patient study number

14a. Are you pregnant? **Olhi mabunda?**

1=yes, 2=no, 3=it is possible, 4=other

14b. If yes at question 14a, refer the paient to the doctor.

15. Do you have any chronic diseases?

Olinha obulwaile obulwirewo?

1=yes 2=no

16. If yes, which diseases?

Oba ihi? bulwaire ki?

17. Do you have diabetes?

Olinha obulwaire bwa sukaali?

1=yes, 2=no, 3= do not know, 4=other (specify)...

18. Do you have a cardiac problem?

Olinha obulwaire bw'omutima?

1=yes, 2=no, 3= do not know, 4=other (specify).......................................
....

19. Do you have any medical condition which affects your lungs?

Olinha obulwaire bwona bwona mu mamawuwe?

1=yes, 2=no, 3= do not know, 4=other
(specify)...

20. Do you have any coagulation disorder?

Olinha obuzibu nti bwe wabawo ekikusaze oba omusaayi okwidha tegulekerawo mangu?

1=yes, 2=no, 3= do not know

21. Do you have any ongoing infectious disease?

Olinha obulwaire obuva mu buwuuka ng'akawuuka akaleeta sirimu, hepatitis oba TB?

1=yes, 2=no, 3= do not know ....

22. If yes, which disease?
Oba yi, bulwaire ki?
1=Hepatitis B, 2=hepatitis C, 3= HIV, 4=TB, 5=other...

23. Do you take any medication regularly?

Olinha amakerenda gomira buli lunaku?

1=yes, 2=no, 3= other ....

24. If yes, which medicine(s)? **Oba yi, makerenda ki?**

1____

2____

3____

4____

25.Have you ever had surgery?

Bakulongoosa ku?1=yes, 2=no, 3= do not know, 4=other (specify).............................

26. If yes, when was it? **Oba yi? Bakulongosaali?**

1= A month ago. 2= More than a month ago? 3= do not know, 4=other (specify)..

27. If yes, which condition was operated?

Oba yi? Bakulongosaaki?

1=groin hernia, 2=do not know, 3=other (specify)..

Patient study number

28. Bwekiba yi 14, bakulongoseza gha?

If yes at question 14, where was the procedure performed

1=Hospital, 2=Other public health facility, 3=Private clinic, 4= Do not know, 5=Other location (specify) ....

29. Oliku n'obuzimbu mu limu oba mu maago gombi?

Do you have a swelling in any or both of your groins?

1=No, 2=Right side, 3=Left side, 4=Both sides, 5=other (Specify)......................

For 30, put an (x) in the appropriate choice.

30. Do you feel pain in any or both of your groins?
Owuliraku obulumi bwonabwona mu limu oba mu maago gombi ?

	Right	Left
1. No pain<br>Wazila bulumi.	□	□
2. Pain present but could easily be ignored.<br>Obulumi buliwo aye busoboka okuguminkirizibwa.	□	□
3. Pain present, could not be ignored, but did not interfere with everyday activities.<br>Obulumi buliwo,tibuguminkirizibwa aye tibundobela kukola milimo edha bulidho.	□	□
4. Pain present, could not be ignored, and interfered with concentration on chores and daily activities.<br>Obulumi buliwo,tibuguminkirizibwa tisobola kwisa isira kumilimo dha bulido.	□	□
5. Pain present, could not be ignored, and interfered with most activities.<br>Obulumi buliwo, tibuguminkilizibwa, bunemesa okukola emilimo	□	□

Patient study number

egisinga bungi.

6. Pain present, could not be ignored, and necessitated bed rest. □ □
   Obulumi buliwo ,tibuguminkilizibwa bumpaliliza kuwumula kukitanda.

7. Pain present, could not be ignored, prompt medical advice sought. □ □
   Obulumi buliwo, tibuguminkilizibwa bumpaliliza kunonya magezi okuva ewomusawo mangu.

Omaze eibangaki ng'owulira obulumi ? □

1=0-6 months, 2=more than 6 months, 3=Don't know

32. Have you had any episodes of severe pain in the groin?

Walikuku n'ebiseera wewawulira obulumi obwamani eihno?

1=Yes, 2=No, 3=Dont know □

33. Additional information and comments

……………………………………………………………………………………………

………………………………………………………………………………………………

………………………………………………………………………………………………

………………………………………………………………………

Appendix 4. Preoperative Physical Examination(for participant)

Patient study number

Filled in by ____

Date and place ____

Identification of the patient

1. Name ____

2. Age in years

Examination

3. Date of examination (dd/mm/yy)

4. Weight (kg)

5. Height (cm)

6. Blood pressure (mmHg/mmHg)

7. Pulse (BPM)

8. Respiratory rate (per minute)

9. Temperature (°C)

10. Heart 1=normal, 2=significant abnormality (specify)……………………………

11. Lungs 1=normal, 2=significant abnormality (specify)

12. Abdomen.1= normal, 2=minor/moderate abnormality (specify

3=scar from previous surgery…………………………………….………………

4=significant abnormality (specify)……………………………………………..

13. Inguinal scar from a previous groin hernia repair

1.Yes 2.No 3. Do not know.

14. Location of inguinal scar *1=N/A, 2=Right side, 3=Left side, 4=Bilateral*

Patient study number

15. Left/right/bilateral *1=N/A, 2=Right side, 3=Left side, 4=Bilateral*

16. Reducible mass *1=N/A, 2=Yes, 3=No*

17. Clinical impression of the groin(s)

1=No groin hernia, 2=Obvious reducible inguinal hernia, 3=Obvous irreducible inguinal hernia,

4=Suspected groin inguinal hernia, 5= Femoral hernia, 7= Suspected Lymphadenitis, 8=Unknown,

10=Other ....

18. Ultrasound examination findings.

i. Groin hernia present?

a. Yes b. no ……………………………………………………………………………

ii. What is the type of groin hernia?

a. Indirect groin hernia. b. direct groin hernia. c. femoral/not specified…………………………………………………….

iii. Size of sac

a. 1-3 cm b.3-5cm c. more than 5 cm

iv. Size of orifice in mm………………………………………………………

v. Bowel content?

a. yes b. no…………………………………………………………………

vi. Completely reducible?

a.yes b. no……………………………………………………………………

vii. Other groin sonography findings.

……………………………………………………………………………………

……………………………………………………………………………………

Patient study number

viii. Sonography examination performed by

……………………………………………………………………………………

19. ASA classification

1= A normal healthy patient, 2=A patient with mild systemic disease,

3=A patient with severe systemic disease,

4=A patient with severe systemic disease that is a constant threat to life,

5=A moribund patient who is not expected to survive without the operation.

20. Recommendation:

1=Hernioplasty, 2=No surgery indicated at present state,

3= Other...

21. Does the patient meet the inclusion and exclusion criteria for the mesh study?

1=yes, 2=no

Inclusion criteria

- Woman
- Age > 18 years
- Reducible inguinal hernia
- Primary inguinal hernia
- Elective inguinal hernia
- The patient accepts to participate
- Ability to give informed consent

Exclusion criteria

- Recurrent hernia
- Anticoagulant medication or coagulation abnormality
- Drug abuse including alcohol with significant Physiological or mental consequences
- ASA group 3 and above
- Known pregnancy

22. Comments:

....
....

23. Parity……………..

24. Ceserean Section 1= Yes 2= No

Appendix 5. Intervention protocol (for participant)

Patient study number

Filled in by ____

Date and place ____

Identification of the patient

1. Name ____

2. Age in years

Intervention

Randomisation
1=A, 2=B

2. Side of operation

1 = Right   2 = Left

3. Time that patient entered operation theatre   hh   mm

4. Start of intervention (first incision)   hh   min

5. End of intervention (last stitch) hh   min

6. Time that patient left the operation theatre   hh   min

7. Hernia type
1=medial, 2=lateral, 3=combined medial and lateral, 4=femoral hernia, 5 =no hernia identified, 5=other…………………………………………………………………

Patient study number

8. Technique used

1= Lichtenstein technique, 2= modified mesh technique, 3= other……………………………………………………………….

9.Cross over

1= Yes   2 No

10. If local anaesthesia was not used or if it was combined with another anaesthetic method, state the reason (if necessary write on backside)

…………………………………………………………………………………………………
…………………………………………………………………………………………………
…………………………………………………………………………

11. Complications during the operation? Specify.

1=Excessive bleeding, assess volume……………………………… ml

2=Accidental damage to other tissue or organ
(specify)………………….………………………………….

3=Injury of staff
(specify)……………………………………………………………………………

4=Anaesthetic complications
(specify)………………………….……………………………………..

5=Other complication
(specify)……………………………………………………………………..

Patient number

Theatre staff

12. Name of surgeon…………………………………………………………………

12. Anesthesia method:
1=local, 2=IV General Anesthesia, 3=Inhalation General Anesthesia, 4=Spinal, 5=Other…………..

13. Title of anesthetist/ position:
1=Self, 2=Anesthetic officer, 3=Other (specify)………………………………………

14. Additional information and comments

……………………………………………………………………………………………

……………………………………………………………………………………………

……………………………………………………………………………………………

……………………………………………………………………………………………

……………………………………

Appendix 6: Operation description for mesh repair method(for participant)

Patient study number

Please describe the procedure you have just performed, from start to end, by filling the following standard questions.

1. Local anesthesia given according to description

2. Skin incision

3. External fascia opened

4. Identification and management of inguinal nerves

	Identified	Isolated	Cut
a.Ilioinguinal nerve			
b.Genital branch of the genitofemoral nerve			
c. Iliohypogastric nerve			

5. Funicle isolated and opened

A. **Lateral hernia**

6. Hernia sack identified and isolated

7. Hernia sack opened

8. Contents of hernia sack. 1=yes, 2=no

9. If yes, 1=omentum, 2=small intestine, 3=large bowel, 4=other (Specify)…………………………..

10. Hernia sack excised

11. Hernia sack invaginated

12. Hernia sack cut and distal part leave in situ

Patient study number ☐☐☐

Femoral hernia.

13. Medial aspect of transversalis fascia opened ☐

14. Femoral canal explored for femoral hernia ☐

15. Femoral hernia identified (Y/N) ☐

16. Hernia sac excised ☐

17. Hernia sac invaginated ☐

18. Mesh cut to fit the patient ☐

19. Slit for coverage of femoral canal cut ☐

20. Mesh put in place. Slit sutured to Inguinal, lacunar and pectineal ligament ☐

21. Suture line in the inguinal floor sutures ☐

22. Mesh sutured according to open anterior mesh method ☐

23. Hemostasis checked ☐

24. External fascia sutured ☐

25. Interrupted skin sutures ☐

Medial hernia

26. Hernia sack identified ☐

27. Hernia sack invaginated by suturing ☐

Patient study number

Femoral hernia

28. Medial aspect of transversalis fascia opened

29. Femoral canal explored for femoral hernia

30. Femoral hernia identified (Y/N)

31. Mesh cut to fit the patient

32. Slit for coverage of femoral canal cut

33. Mesh put in place. Slit sutured to inguinal
    Lacunar and pectineal ligament.

34. Suture line in the inguinal floor sutured

35. Mesh sutured according to Lichtenstein method

36. Hemostasis checked

37. Mesh sutured according to Lichtenstein method

38. External fascia sutured

39. Interrupted sutures of the skin

37. No hernia sack identified (add comments below)

38. Additional information and comments

____

____

____

Appendix 7. Discharge form(for participant)

Patient study number

Filled in by ____

Date and place (dd/mm/yy) ____________________

Identification of the patient

1. Name ____

2. Age in years

3. Discharge date

4. If the patient was not discharged the same day as the surgery, specify the reason.

……………………………………………………………………………………………

……………………………………………………………………………………………

……………………………………………………………………………………………

……………………………………………………………………………………………

……………………………………………………………………………………………

……………………………………………...

Appendix 8. Follow up 2 weeks(for participant)

Patient study number

Filled in by ______________________________

Date and place ______________________________

Identification of the patient

1.Name ______________________________

2. Name of district ______________________________

3. Village name ______________________________

4. Mobile phone number 1 ______________________________

5. Mobile phone number 2 ______________________________

6. Mobile phone number 3 ______________________________

7. Local chairman ______________________________

8. Phone number of local chairman ______________________________

9. Occupation ______________________________

10. Place of work (if any) ______________________________

11. Additional contact information ______________________________

____

____

____

Patient study number

Follow up interview (for participant)

12. Have you had any complications or problems after the hernia surgery?

Wafunyeku obuzibu bwona bwona ng'omaze okulongosebwa?

1=yes, 2=no, 3=other

(specify)...

13. If yes, which kind of problem(s) did you have?..

1= pain, more than expected which demanded more pain killers/additional or exchange of painkillers, 2=Bleeding; discoloration of the skin around the wound or the labia 3=bleeding; significant swelling and tension of the skin, not only miscoloration, 4=Infection; which needed antibiotics, 5= Infection which demanded that the wound be opened, 6=significant problem to urinate; with need for catheterization, 7=Pain in labia; the same side as the operated hernia, 8=embolus (blood clot) in leg or lung, 9=pneumonia, 10=Cardiac infarction, 11= Recurrence of the hernia, 12=Other (specify)

14. If there was a problem or complication, how severe was it?

Obuzibu bwe wafunye, bubaire bwaghaha? ....

1=Tibwamani einho (mild), 2=intermediate/moderate,
3=Bwamani (severe), 4= Buyinza okusanya w'obulamu (life threatening)

15. Are you satisfied with the result of your surgery so far?

Olimusanufhu ne biviremu mu kulongesebwa?

1=yes, 2=no, 3=other____

Patient study number

16. Have you sought and/or accessed health care for the hernia/wound after the surgery?

Wafunyeku obwindadhabi bwa hania oba obwe kiwundu obundi ngomaze okulongosebwa

1=yes, 2=no

17. Would you recommend a friend or relative to be operated for hernia under local anaesthesia?

Osobola okusikiriza mukwanogwo oba owo luganda okulongosebwa hania nga basanalaziza hania yonka?

1=yes, 2=no, 3=other____

18. Do you think that we should continue to perform hernia surgery as day case surgery?

Olowoza nti okulongoosa hania kugye mu maiso nga abalwaire baidha kwolwo, memale bairayo eka?

1=yes, 2=no, 3=other____

19. Other comments and information

Waliwo ekindi kyoyenda okukoba?

…………………………………………………………………………………………

…………………………………………………………………………

…………………………………………………………………………………………

…………………………………………………………………………

Patient study number

Date (dd /mm/ yy)

Physical examination (for participant)

20. Clinical examination

1. Operation wound healed properly, 1=yes, 2=no
2. Remaining discomfort, 1=yes, 2=no

   If yes, in what way? …………………………………………………………
3. Recurrent hernia, 1=yes, 2=no
4. Other……………………………………………………………………………

   ……………………………………………………………………….

21. Complications after the surgery?

1=None, 2=miscoloration of the skin,
3=hematoma, 4=superficial infection, 5=deep infection, 6=severe palpatory pain in the groin, 7=other…………………………………

22. If there is a problem or complication, how severe is it?

1=Mild, 2=intermediate/moderate, 3=severe, 4= life threatening

**Complication**	**Definition**
Hematoma	Bleeding causing significant tissue distension, bruising excluded
Urinary retention	Need for catheterisation
Infection	Local signs of inflammation, purulent secretion and/or positive wound culture
Severe pain	Out of the expected postoperative pain
Other complication	seroma, thromboembolism, cardiovascular, infections other than in the wound
Need for reoperation, other than recurrence	
Death	

Appendix 9. Follow up 1 year

Patient study number

Filled in by ______________________________________

Date and place ____________________________________

Identification of the patient

1. Name ____
2. Name of district ______________________________________
3. Village name ____
4. Mobile phone number 1 _________________________________
5. Mobile phone number 2 _________________________________
6. Mobile phone number 3 _________________________________
7. Local chairman _______________________________________
8. Phone number of local chairman ___________________________
9. Occupation ____
10. Place of work (if any) __________________________________
11. Additional contact information ____________________________

____

Patient study number

The following questions regard the pain that you have now or have had in your groin during the past week.

12. Tebeleza obulumi obusingha amaani bwowulile mu maago week ebise

Estimate the <u>worst pain</u> you have felt in the <u>operated groin</u> during this past week.

1. No pain □
   Wazira bulumi.
2. Pain present, but can easily be ignored □
   Obulumi buliwo, aye tibufaaku.
3. Pain present, cannot be ignored, but does not interfere with everyday activities □
   Obulumi buliwo , mbuwulira aye tibundobera kukola

   mirimu ya bulidho.
4. Pain present, cannot be ignored, interferes with concentration on chores and daily activities. □
   Obulumi buliwo, tibundhikiriza kukola mirimu yange bulidho.
5. Pain present, cannot be ignored, and interferes with most activities. □
   Obulumi buliwho, bundobera okukola emirimu yange bulidho.
6. Pain present, cannot be ignored, and necessitates bed rest. □
   Obulumi buliwho, mba mukitanda buli kiseera.
7. Pain present, cannot be ignored, prompt medical advice sought. □
   Obulumi buliwho, nafunyeku obwindhandhabi.

If the patient answered no pain in question 12, please continue with question 13 and then questions 17 -20

13. If you answered “no pain” to question 10 above, try to remember when the pain in the operated groin disappeared after the operation.

1. The pain in the operated groin disappeared within 1 month after the operation. □
   Obulumi mu luuyi olwa longosebwa bwagya mu mwezi mulala.
2. The pain in the operated groin disappeared 1-3 months after the operation. □

Patient study number

☐ ☐

Obulumi mu luuyi olwa longoseebwa lwagya wagati womwezi mulala ne satu.

3. The pain in the operated groin disappeared 4-6 months after the operation.
   Obulumi mu luuyi olwalongoseebwa bwaja mu myezi inha n’omukaaga.

4. The pain in the operated groin disappeared 7-12 months after the operation. □
   Obulumi mu luyi olwalongoseebwa bwaja mu myezi musanvi n’omwaka

5. The pain in the operated groin disappeared recently. □
   Obulumi mu luyi olwalongoseebwa bwakaja butibuti.

□

If the patient has felt pain in the operated groin *during the past week*, please answer question 14 and 15.

14. How often have you felt pain in the operated groin during the past week?

Obulumi mu luuyi olwa longoseebwa obaire obuwulira otya?

1. Once a week. □
   Mulundi mulala mu sabiiti
2. 2-5 times a week. □
   Emirundi ng’ebiri ku etaano mu sabiiti
3. Every day. □
   Buli lunaku mbuwulira
4. Every day and also during night time. □
   Buli lunaku omusana ne kiro
5. I have had pain the whole week, both day and night □
   Obulumi mbuwulira sabiiti yona yona omusana ne kiro.

15. Have you taken pain-killers for pain in the operated groin during the past week?

Wamizeko amakerenda gobulumi ngomaze okulongosebwa sabiiti ebiseho?

1=no, 2=yes, 3=other.. ☐

Patient study number

16. To what extent has pain in the groin limited your working capability/capacity to perform daily activities in the last 2 months?

Nenda okugerageranya obunene bw'obulumi bwo baire owulira nobusobozi bwo kukola emirimo yo buli lunaku.

1. I have been performing my normal activities as usual □
   Emirimo yange mbaire ngikola bulungi buli lunaku
2. The pain made me abstain from normal activity 1-7 days during the last 2 months □
   Obulimi bwandobera okukola emirimu yange wagati yolunaku lulala n'omusanvu mu myezi ebiri ebiseeho.
3. The pain made me abstain from normal activity 1-4 weeks during the last 2 months □
   Obulumi bwandobera okukola wagati ya sabiiti ndala ku inha mu myezi ebiri ebiseho.
4. The pain has made me abstain from normal activity for the whole of the last 2 months □
   Obulumi bwandobeire okukola okumala emyezi ebiri yona yona ebiseho.

17. How is it today with pain and other complaints in the operated groin compared?

to the pain and complaints before the operation? Is it the same, more or less?

Obulumi bulibutya buti ngo geraganya nebibaire bikuluma ku luyi olwa

longoseebwa. Obulumi nobwo, bukendeire obwa bweyongeire. □

1=Less, 2=The same, 3=More

18. Are you fully recovered in the operated groin? □

Owonye bulungi ku luuyi olwa longoseebwa

1=yes, 2=no, 3=other

Patient study number

19. If no, which remaining problems do you have?

Bwoba okaali kuwona ekindi ki?

1=Pain, 2=limited physical movement, 3=numbness,

20. Are you satisfied with the results of your hernia surgery?

Oli musanafu nebivireemu mu kulongosebwa?

a.Yes, b. No

21. Have you been operated on for hernia or had an abdominal operation since the hernia operation at ________________________ ____Hospital in 20____?

Walongoseebwako hania or okulongosebwa ku nda ngomaze

okulongesebwa hania?

1=yes, 2=no, 3=other...

Patient study number

Date (dd /mm/ yy)

Physical examination (for participant)

22. Clinical examination

5. Operation wound healed properly, 1=yes, 2=no
6. Remaining discomfort, 1=yes, 2=no
   If yes, in what way? …………………………………………………………
7. Recurrent hernia, 1=yes, 2=no
8. Other…………………………………………………………………………………
   ………………………………………………………………………….

23. Complications after the surgery?

1=None, 2=miscoloration of the skin,
3=hematoma, 4=superficial infection, 5=deep infection, 6=severe palpatory pain in the groin, 7=other…………………………………

24. If there is a problem or complication, how severe is it?

1=Mild, 2=intermediate/moderate, 3=severe, 4= life threatening

**Complication**	**Definition**
Hematoma	Bleeding causing significant tissue distension, bruising excluded
Urinary retention	Need for catheterisation
Infection	Local signs of inflammation, purulent secretion and/or positive wound culture
Severe pain	Out of the expected postoperative pain
Other complication	seroma, thromboembolism, cardiovascular, infections other than in the wound
Need for reoperation, other than recurrence	
Death	

Patient study number

25. Other comments and information

……………………………………………………………………………………………

……………………………………………………………………………………………

……………………………………………………………………………………………

……………………………………………………………………………………………

……………………………………………………………………………………………

……………………………………………………………………………………………

……………………………………………………………………………………………

……………………………………………………………………………………………

……………………………………………………………………………………………

……………………………………………………………………………………………

……………………………………………………………………………………………

……………………………………………………………………………………………

……………………………………………………………………………………………

……………………………………………………………………………………………

……………………………………………………………………………………………

Examination by (name + signature) ______________________

Appendix 10. Follow up after 3 years

Patient study number

Filled in by ______________________________

Date and place ______________________________

Identification of the patient

1. Name ______________________________

2. Name of district ______________________________

3. Village name______________________________

4. Mobile phone number 1 ______________________________

5. Mobile phone number 2 ______________________________

6. Mobile phone number 3 ______________________________

7. Local chairman______________________________

8. Phone number of local chairman ______________________________

9. Occupation______________________________

10. Place of work (if any) ______________________________

11. Additional contact information ______________________________

____

Patient study number

The following questions regard the pain that you have now or have had in your groin during the past week.

12. Tebeleza obulumi obusingha amaani bwowulile mu maago week ebise

Estimate the worst pain you have felt in the operated groin during this past week.

8. No pain ☐
   Wazira bulumi.
9. Pain present, but can easily be ignored ☐
   Obulumi buliwo, aye tibufaaku.
10. Pain present, cannot be ignored, but does not interfere with everyday activities ☐
    Obulumi buliwo , mbuwulira aye tibundobera kukola

    mirimu ya bulidho.
11. Pain present, cannot be ignored, interferes with concentration on chores and daily activities. ☐
    Obulumi buliwo, tibundhikiriza kukola mirimu yange bulidho.
12. Pain present, cannot be ignored, and interferes with most activities. ☐
    Obulumi buliwho, bundobera okukola emirimu yange bulidho.
13. Pain present, cannot be ignored, and necessitates bed rest. ☐
    Obulumi buliwho, mba mukitanda buli kiseera.
14. Pain present, cannot be ignored, prompt medical advice sought. ☐
    Obulumi buliwho, nafunyeku obwindhandhabi.

Patient study number

If the patient answered no pain in question 12, please continue with question 13 and then questions 17 -20

13. If you answered "no pain" to question 10 above, try to remember when the pain in the operated groin disappeared after the operation.

6. The pain in the operated groin disappeared within 1 month after the operation. □
   Obulumi mu luuyi olwa longosebwa bwagya mu mwezi mulala.

7. The pain in the operated groin disappeared 1-3 months after the operation. □
   Obulumi mu luuyi olwa longoseebwa lwagya wagati womwezi mulala ne satu.

8. The pain in the operated groin disappeared 4-6 months after the operation.
   Obulumi mu luuyi olwalongoseebwa bwaja mu myezi inha n'omukaaga. □

9. The pain in the operated groin disappeared 7-12 months after the operation. □
   Obulumi mu luyi olwalongoseebwa bwaja mu myezi musanvi n'omwaka

10. The pain in the operated groin disappeared recently. □
    Obulumi mu luyi olwalongoseebwa bwakaja butibuti.

Patient Study number

If the patient has felt pain in the operated groin *during the past week*, please answer question 14 and 15.

14. How often have you felt pain in the operated groin during the past week?

Obulumi mu luuyi olwa longoseebwa obaire obuwulira otya?

6. Once a week. □
   Mulundi mulala mu sabiiti
7. 2-5 times a week. □
   Emirundi ng'ebiri ku etaano mu sabiiti
8. Every day. □
   Buli lunaku mbuwulira
9. Every day and also during night time. □
   Buli lunaku omusana ne kiro
10. I have had pain the whole week, both day and night □
    Obulumi mbuwulira sabiiti yona yona omusana ne kiro.

15. Have you taken pain-killers for pain in the operated groin during the past week?

Wamizeku amakerenda gobulumi ngomaze okulongosebwa sabiiti ebiseho?

1=no, 2=yes, 3=other..

Patient study number

16. To what extent has pain in the groin limited your working capability/capacity to perform daily activities in the last 2 months?

Nenda okugerageranya obunene bw'obulumi bwo baire owulira nobusobozi bwo kukola emirimo yo buli lunaku.

1.I have been performing my normal activities as usual □
Emirimo yange mbaire ngikola bulungi buli lunaku

2.The pain made me abstain from normal activity 1-7 days during the last 2 months □
Obulimi bwandobera okukola emirimu yange wagati yolunaku lulala n'omusanvu mu myezi ebiri ebiseeho.

3.The pain made me abstain from normal activity 1-4 weeks during the last 2 months □
Obulumi bwandobera okukola wagati ya sabiiti ndala ku inha mu myezi ebiri ebiseho.

4.The pain has made me abstain from normal activity for the whole of the last 2 months □
Obulumi bwandobeire okukola okumala emyezi ebiri yona yona ebiseho.

17. How is it today with pain and other complaints in the operated groin compared?

to the pain and complaints before the operation? Is it the same, more or less?

Obulumi bulibutya buti ngo geraganya nebibaire bikuluma ku luyi olwa longoseebwa. Obulumi nobwo, bukendeire obwa bweyongeire.

1=Less, 2=The same, 3=More

18. Are you fully recovered in the operated groin?

Owonye bulungi ku luuyi olwa longoseebwa

1=yes, 2=no, 3=other

19. If no, which remaining problems do you have?

Patient study number

Bwoba okaali kuwona ekindi ki?

1=Pain, 2=limited physical movement, 3=numbness,

20. Are you satisfied with the results of your hernia surgery?

Oli musanafu nebivireemu mu kulongosebwa?

a.Yes, b. No

21. Have you been operated on for hernia or had an abdominal operation since the hernia operation at _______________________ ____Hospital in 20____?

Walongoseebwako hania or okulongosebwa ku nda ngomaze

okulongesebwa hania?

1=yes, 2=no, 3=other...

Patient study number ☐☐☐

Date (dd /mm/ yy) ☐☐☐☐☐☐

Physical examination

22. Clinical examination

9. Operation wound healed properly, 1=yes, 2=no ☐
10. Remaining discomfort, 1=yes, 2=no ☐
    If yes, in what way? ……………………………………………………………
11. Recurrent hernia, 1=yes, 2=no ☐
12. Other……………………………………………………………………………
    ……………………………………………………………………………………..

23. Complications after the surgery? ☐☐☐☐☐

1=None, 2=miscoloration of the skin,
3=hematoma, 4=superficial infection, 5=deep infection, 6=severe palpatory pain in the groin, 7=other…………………………………

24. If there is a problem or complication, how severe is it? ☐

1=Mild, 2=intermediate/moderate, 3=severe, 4= life threatening

**Complication**	**Definition**
Hematoma	Bleeding causing significant tissue distension, bruising excluded
Urinary retention	Need for catheterisation
Infection	Local signs of inflammation, purulent secretion and/or positive wound culture
Severe pain	Out of the expected postoperative pain
Other complication	seroma, thromboembolism, cardiovascular, infections other than in the wound
Need for reoperation, other than recurrence	
Death	

Patient study number

25. Other comments and information

……………………………………………………………………………………………

……………………………………………………………………………………………

……………………………………………………………………………………………

……………………………………………………………………………………………

……………………………………………………………………………………………

……………………………………………………………………………………………

……………………………………………………………………………………………

……………………………………………………………………………………………

……………………………………………………………………………………………

……………………………………………………………………………………………

……………………………………………………………………………………………

……………………………………………………………………………………………

……………………………………………………………………………………………

……………………………………………………………………………………………

……………………………………………………………………………………………

Examination by (name + signature) ______________________

Patient study number

Appendix 11. Post operative information for patients

Expected symptoms and recommendations after the surgery.

Obubonero obusuubilwa n'ebinakolebwa nga okulongoosebwa kuweire.

- After the surgery you will feel some pain. Diclofenac, Ibuprofen and paracetamol will be sufficient in reducing this pain most of the time. You should not use Aspirine or Diclofenac before surgery since they increase the risk for bleeding.

  Ng'omaze okulongoosebwa, oidha kuwulira muku obulumi butono. Panado aida kuba nga amala okukendeezaku obulumi bunno ebiseera ebisinga. Totekeirwa kukozesa asupilini oba dikulofenaka okuva lwebili nti byongera ku buzibu obw'okuduludha omusaayi.

- Because of the pain, you may not be able to perform all normal activities during the first 10-14 days after the surgery. However, it is important to be physically active and move around as usual.

  Kulw'obulumi,oyinza obutasobola kukola mirimo gyo egyabulidho mu sasira/wiki 10-14 edhisooka nga wakalongoosebwa. Wabula, kyamugaso inho okuba ng'okozesa omubili gwo era otambule tambuleku agho nga buliidho.

- Small bleedings in the skin are common. They may discolor the wound area and may also give a discoloration of the scrotum. This is harmless and it will disappear within 30 days.

  Okusabuka sabuka okutono mu luwusu bitera okubaagho. Biyinza okukyuusa langi agho waibwa era biyinza n'okukyuusa langi y'akasagho ak'enkulo (amaye). Kino kizira bulabe era kiidha kughawo munaku 30.

- The scar will be somewhat thick and hard during the first months after the surgery. With time, the scar softens.

  Enkovu eidha kuba nnene muku era nga ngumu mumyezi egisooka nga wakalongoosebwa. Nga wabise akaseera, enkovu egonda

Patient study number

- Numbness around the scar is common but harmless.

  Okusanhalala w'enkovu kitera okubaagho aye kizira bulabe.

- The sutures will be removed at the post operative control after 14 days. At the same visit, you will be seen by a medical doctor for physical examination in order to find complications at an early stage.

  Ewuuzi diidha kutoolebwagho…………………………..Ku lukyala olulala olwo,oidha kubonebwa omusawo ow'ebyobulamu aidha okukebera abonhe nti azura obuzibu nga bwakatandika.

Possible complications that need medical attention. Obuzibu obutono tono obwetagisa okubonebwa oweby'obulamu.

- Infection in the wound. The wound becomes very swollen and painful and sometimes pus drains from the wound. *Obulwaire mu kiwundu. Ekiwundu kizimba inho era kyaruma n'ebiseera ebindhi amasira gakulukuta okuva mu kiwundu.*
- Hematoma. Significant bleeding under the skin so that the wound and/or scrotum becomes swollen
- If you experience these or any other problems after the surgery, we encourage you to contact Dr Alphonsus Matovu, phone number 0774 287 185 to discuss the problems and plan an extra check up if necessary.

The planned control after 14 days will take place the ………………………(date)

at …………………………..(time) at ……………………… (Location)

by Dr. ………………………………………………………….

Patient study number

Appendix 12:WHO Surgical safety check list.

Surgical Safety Checklist

World Health Organization | Patient Safety
A World Alliance for Safer Health Care

Before induction of anaesthesia

(with at least nurse and anaesthetist)

Has the patient confirmed his/her identity, site, procedure, and consent?
- ☐ Yes

Is the site marked?
- ☐ Yes
- ☐ Not applicable

Is the anaesthesia machine and medication check complete?
- ☐ Yes

Is the pulse oximeter on the patient and functioning?
- ☐ Yes

Does the patient have a:

Known allergy?
- ☐ No
- ☐ Yes

Difficult airway or aspiration risk?
- ☐ No
- ☐ Yes, and equipment/assistance available

Risk of >500ml blood loss (7ml/kg in children)?
- ☐ No
- ☐ Yes, and two IVs/central access and fluids planned

Before skin incision

(with nurse, anaesthetist and surgeon)

- ☐ **Confirm all team members have introduced themselves by name and role.**
- ☐ **Confirm the patient's name, procedure, and where the incision will be made.**

Has antibiotic prophylaxis been given within the last 60 minutes?
- ☐ Yes
- ☐ Not applicable

Anticipated Critical Events

To Surgeon:
- ☐ What are the critical or non-routine steps?
- ☐ How long will the case take?
- ☐ What is the anticipated blood loss?

To Anaesthetist:
- ☐ Are there any patient-specific concerns?

To Nursing Team:
- ☐ Has sterility (including indicator results) been confirmed?
- ☐ Are there equipment issues or any concerns?

Is essential imaging displayed?
- ☐ Yes
- ☐ Not applicable

Before patient leaves operating room

(with nurse, anaesthetist and surgeon)

Nurse Verbally Confirms:
- ☐ The name of the procedure
- ☐ Completion of instrument, sponge and needle counts
- ☐ Specimen labelling (read specimen labels aloud, including patient name)
- ☐ Whether there are any equipment problems to be addressed

To Surgeon, Anaesthetist and Nurse:
- ☐ What are the key concerns for recovery and management of this patient?

This checklist is not intended to be comprehensive. Additions and modifications to fit local practice are encouraged.

Revised 1 / 2009

© WHO, 2009

Appendix 13: Adverse event classification and reporting tool.

(Adapted from PEPFAR's best practices for Voluntary Medical Male Circumcision site operations; Chapter 7. Managing, Monitoring, Reporting VMMC Adverse events).

Encircle as appropriate

Participant number

Adverse event	Mild	Moderate	Severe
AN. Anaesthetic related problem			
Surgery	Mild localized allergic reaction at injection site without swelling and allergic reaction	Symptoms of reaction to anesthetic including light headedness, nervousness or dizziness. These symptoms may resolve on their own and may not necessitate use of emergency commodities such as medicines or equipment from the emergency kit. These symptoms do not require admission to hospital or transfer to another health facility.	Symptoms of severe systemic allergic reaction to local anesthetic including rash, urticaria, angiooedema and shortness of breath, or symptoms of overdose of local anaesthetic including light headedness, nervousness, confusion, dizziness, drowsiness, ringing of ears, blurred or double vision, sensations of heat, cold or numbness, twitching, tremors, convulsions, unconsciousness, respiratory depression, braddycardia or hypotension requiring use of medicines or equipment from the emergency kit, or emergency commodities or hospitalization to

			manage.
BL. Bleeding			
Surgery	Intra operative bleeding that is more significant than usual or post operative spotting of the bandage with blood, both easily controlled.	Intraoperative bleeding or bleeding that occurs prior to discharge that requires a pressure dressing to control or that requires additional skin sutures without surgical re-exploration of the wound.	Intraoperative bleeding requiring blood transfusion, transfer to another facility, or hospitalization or transfer to another facility.
PA. Pain			
Surgery	Client expresses discomfort, however is able to remain still and co-operate for the procedure. No additional local anaesthetic is required.	Pain requiring additional local anesthesia	Pain not responsive to additional local anesthesia.
POST OPERATIVE PERIOD			
BL. Bleeding			
Surgery	Blood stained dressing or underwear, no active bleeding. Small amount of bleeding from minor clot disruption when changing dressings that is controllable with new dressing or within 5-10mins of manual pressure	Bleeding that is not controlled by a new dressing or within 5-10mins of manual pressure measured on a clock, or requires return to the health facility for a pressure dressing or additional skin sutures without surgical re-exploration of the	Bleeding that requires surgical re-exploration, hospitalization or transfer to another facility or any case where blood transfusion or iv, fluids is necessary.

	measured on a clock.	wound	
IN. Infection			
Surgery	Erythema or traces of serous discharge or infective process noted at wound margin. No intervention required other than observing wound hygiene.	Discharge from the wound, painful swelling with erythema, or elevated temperature requiring the use of oral antibiotics.	Cellulitis or abscess of the wound or infection severe enough to require surgical intervention, hospitalization, intravenous or intramuscular antibiotics.
PA. Pain			
Surgery	Participant complains of pain , not requiring more than standard post operative analgesia and considered within normal thresholds	Pain serious enough to result in disability (as evidenced by loss of work or cancellation of normal activity. On a Visual analogue scale it is scored 5-7 on a scale of 1-10.	Pain serious enough to result in disability (as evidenced by loss of work or cancellation of normal activity. On a Visual Analogue Scale it is scored 8-10 on a scale of 1-10.

Appendix 14: Costing of groin hernia repair methods

Participant number

SN	Types of medicines used	Amount in mls or gms	Cost on UGX	Cost in USD
a	Flucloxacillin	1.5gms		
b	Clindamycin	900gms		
c	Lidocaine			
d	Ropivacaine			
e	Paracetamol			
f	Ibuprofen			
g	Mesh			
h	Sutures<br>i.<br>ii.<br>iii.<br>iv.<br>v.<br>vi			
	**Cost of the operation**	**Quantity**	**Cost in UGX**	**Cost in USD**
a	Cost of procedure			
b	Cleaning of operating rooms			
c	Cleaning of equipment			
c	Sterilisation of equipment			

e	Cleaning of linen			
f	Sterilization of the linen			
	**Human resource costs**			
a	Surgeon			
b	Medical officer			
c	Scrub nurse			
d	Instrument nurse			
e	Ward nurse			
Top up administrative costs				
g	Electricity			
h	Water			
	**Additional information**			

Appendix 15: Work plan for the study.

**Work plan for female groin hernia surgery study.**			
**Serial number**	**Activity**	**Date**	**Persons responsible**
1	Completing study documents	September-2018	Matovu
2	Submission of study documents	October –November 2018	Matovu
3	Submission to UNCST	November -2018	Matovu
5	Processing Practicing Licenses for visting investigators	November -2018	Matovu
6	Mobilisation of participants	January 2019	Matovu
7	Training surgeons	February-2019	Pär, Andreas, Alphons, Jenny
8	Continuing surgeries for participants	February to May 2019	Matovu
9	2 week follow up of participants	February to June 2019	Matovu
10	Data entry and filtering	July-2019	Matovu
10	One year follow up of participants	February to June 2020	Matovu
11	Completion of data entry	March-2020	Matovu
12	Three year follow up	February to May 2022	Matovu
12	Writing manuscript	Ongoing	Matovu, Jenny, Pär, Andreas

Addendum 1: Endagano yo kwikiriza mu Lusoga. Lusoga consent form

Enamba ye yenhigire mukunhonereza

Enandhula: Erinha lyange nhi nze…………………………………… era ndi mulala kwabo abali kunhonhereza ku musomo ogutuumibwa okugerangeranya wagati wokulongoosa kwe mpalama okwenkola eyokusala bataamu akatimba nokulongoosa okwenkola erikugezebwa eyokusala bataamu akatimba mu bakazi aye nga bakyusizaamu engeri ye bakataamu. Okulongoosa kuno kwidha kuba kwa mirundi ebiri era nga tugezesa enkola edho dhombi. Okulongoosa kuno kwakasibwa akakiiko akaikirizibwa nga kakagema ku nonhereza edhe dhawulo aketebwa Mildmay Uganda Research and Ethics Committee.

Omugaso:

Empalama (inguinal Hernia) bulwaile bumanhibwa munsi yonayona eramukunhonhereza okuweire, abasadha ikumi kubuli kikumi (10%) balinaye.Engeri enangu gyebakozesa okulongosa empalama (hernia) eno mubasadha yali ya kwisamu katimba, kawanhirire obuzito bwe bitundu ebifulwime ela katangire obulwaire obutairamu. Ate mu bakyala, okunhonhereza kubaire kutono okutegera engeri esinga okozesebwa mu kulongosa empalama eyo. Aye kiri mubwerufu nti enongosa gye batera okukozesa mubasadha tematiza ku luyi olwabakazi.Mukunhonhereza okuweire okwakolebwa kunongosa ye empalama mu basadha,twazula nti akatimba akesente entono,kali kalungi ate nga kesigika.Mukunonereza kuno twida kuzula oba akatimba kasobola okozesebwa ni ku bakazi nga tutairemu obukugu obwetagisibwa ku balwaire bakazi.Tuja kugerangeranya enkola eyo nedhiliwo empyaka dhona nga dhikozesa katimba.

Emitendera jokunhonhereza:

Okunhonhereza kunho kuja kwetabamu abakazi 440 abali nhe empalama(inguinal hernia) okuva mubifo byetwalonda okuli, Mubende Regional Referral hospital, Iganga hospital, Hoima Regional Referral Hospital, Jinja Regional Referral Hospital, Buluba hospital, Kitovu Hospital, Tororo Hospital, Kitgum Hospital, Lacor Hospital, Arua

Regional Referral Hospital and Kamuli Mission Hospital. Ekitundu ku bakazi baidha kulongosebwa munkola ebangawo oba enkaile ate ekitundu ekindi kilongosebwe munkola enongosemu era ekanoonherezebwaku. Abalwaire tibaidha kumanha nongosa eyabakoleibwaku. Okulongosebwa kwidha kolebwa abakugu mu kulongosa, nga basose kusanhalaza ekitundu kyokulongosebwa. Kino kitegeza nti omulwaire aidha kuba ategera nga bamulongosa era nga tawulira bulumi.Okulongosa kwidha kutwala wagati wadakika 60 ne 120.Nga okulongosa kuweire, omulwaire aidha kuba asobola okutambula bulungi era tikidha kuba kyabulabe okuja eka kulunaku lwene olwo.Nga wabise wiiki ibiiri, omwaka mulala, ne myaka esatu, omulwaire aidha kubuzibwa ebibuzo ate omusawo (doctor) amale amukebere okutegera ebyava mukulongosa. Aba ne nhigira mu kunonhereza kuno baidha kuwebwa amawulire agagema ku ntambula yokunhonhereza nebinaaba bizuulibwa buli lwe banairanga okukeberebwa.

Emiganhulo mu kunhonhereza: Oja kulongosebwa empalama kubwerere. Oidha kufuna emitwalo ebiri (20,000sh) dhikuyambeku muntambula buli lwonaidha ngha okukyala, oba okutegezebwa ebigemagana nokunhonhereza. Katugeze nga wa kaingizibwa, nga omaze wiiki ibiri nga oingizibwa,nga oize okeberebwa, nga omaze omwaka mulala ogwokwenhigiramu, nga era omaze emya esatu nokukeberebwa okusembayo nga emyaka esatu gyiweireyo.

Bwofuna obuzibu obwekusa kukulongosebwa, twidha kukuvunanizibwaku ela tukwidhandhabe bulungi paka nga otereire. Oidah kunhonhezebwaku nga wabisewo wiiki ibiri, no mwaka mulamba tusobole okutegera obuvunhe obunakutukaku era tukwidandabe mu bwiire.

Ebizibu ebisubirwa mukunhonhereza:

Okulogosebwa kwonakwonw kwidha ne bizibu ebitontono tugeze nga, abalwaire abandi bafuna obulumi nga bamaze okulongosebwa aye batono kwabo abasigala nobulumi okumala eibanga. Abandi bavamu omusayi mungi mu kiwundu , waliwo no kufuna obuwuka obuleta amasila okuva mu kiwundu. Mu balwaire abandi, empalama esobola okwira. Bwoba ofunye

ekilala kubuvunhe obwo bwemenye, manisa omukulu wokunhonhereza kuno ate tambulilawo oire we twakolera okunhonhereza ofunhe

Enamba ye yenhigire mukunhonereza

obuyambi. Ebyerarikiriza ebiva mubuvunhe bidha kendezebwa ne enkola enungamu okuva mu bakulu bokunhonhereza.Abalwaire batono abairamu okufuna hania ate bwekinabawo, twidha kwiramu tukulongose kubwerere.

Okwenda okwenhigiramu:

Okwenhigira mukunhonhereza kuno kwakyeyendele. Bwoba olonze oba omulala kwabo abaja okwenhigiramu,nja soka nkusabe onsinhingireku oba oteku ekinkumu kukiwandhiko kinho okukakasa nti oikiliza okwenhighira mu kunhonhereza kuno**.**

Nolwekyo, nja kukubuza ebibuzo ebigemagana kubulamubwo mukutwaliza awalala ni hania okusingila ilala. Walala twidah kusalawo olunaku lwonaidha mu dwaliro elikuli okumpi okulongosebwa. Baidha kukulongosa lunaku olunairaku. Omusawo aidha kukukebera okubona bwoli nga wabisewo wiiki ibiiri, omwaka mulala ne myaka esatu nga omaze okulongosebwa.

Okuuma ebyama:

Byoja okutukobera bija kukumibwa bulungi ela tiwaidhakuba muntu yena yena aida kubitegera okutolaku abakulu abakulembairemu okunhonhereza kuno bonka. Byona byona ebikumhanhisa nga kwotaire ne erina lyo bidha kwekebwa era tibiragibwe muntu yena yena mukwekenenya nhi mukusansanya ebinava mukunhonhereza. Bwoba oli nekibuzo ekigemagana kukunonhereza kuno bambi mbuza, oba akulembeiremu okunhonhereza kuno, Dr. Matovu Alphonsus , 0774287185, osobola omwebuzaku ku bibuzo ebyendawulo. Kusalawo kwo okwikiriza okwenhigira mukunhonhereza ate oli waidhembe okuvamu ekisera kyonakyona era kizira mbolodanye dekileta wagati waife nhighe.

Ebibuzo ebyekusa ku idhembe elye yenhigiremu, oba okwemulugunya ku kunhonhereza kuno disobola okusidikibwayo mukyala gwebeta Ms Harriet Chemusto ku namba 0392-174-236 akulira ekitongole kyebeta Mildmay Uganda Research and Ethics Committee. Okugaita

kwebyo eyenighire mu ku nonhereza asobola okutukirira omukulembeze wakakiiko akebyenonhereza okumanha ebisingawo kwi idhembe lye ne byendabirira.

Enamba ye yenhigire mukunhonereza

Okwikiriza

Nga tukali kweyongelayo na bibuzo, Nandyenze okusaba olukusa, oikiliiza okwenhigira mu kunhonhereza kuno?

Nnhinonolebwa bulungi ebigemagana nhokunhonhereza kuno era ntegeire omugaso nebigendhelelwa.Ntegeire byonabyona ebigigugemaku era ntegezeibwa nebyetagisibwa mu kunhonhereza kuno. Ebibuzo byange babizemu namatira. Ndikiliza okwenhigira mu kunhonhereza.

Elina lye ye nhigilemu…………………………………………………

Sain/ ye yenhigilemu…………………………………………………..

Enaku dho mwezi……………………………………………………..

Ekinkumu kye yenhigilemu……

Elina lya bairewo oba eyenhigilemu tasobola kusaininga…………………………

Sain ya bairewo………………………………………

Enaku dho mwezi…………………………………….

Elina lya abuuza……………………………………………………..

Sain ya abuuza……………………………………………………..

Enaku dho mwezi……………………………………………………..

1869

1870

1871

Addendum 2 :Acholi translation.

ORIGWAN. 1. **Number pa latwo**

Me acel: Ayee ni gini timere.

Nyute: Nyinga obedo …………………………… an abedo dul ajo ame atye akwedo kop kwan man ame abino bari lakwot. Kom ikin mon. Ikin datari madit kwede . Latwo pe ngeyo kwan man obin omoko dul. Madit malyo obin omoko olwongo ni (Mildmay Uganda)

Pire. Two ni mako dano mapol iwi lobo ni weng,ikin coo madongo ku nyango me UGANDA, gi nwongo ni tye apar(10%) ikom miacel(100%). Ikwan mu kato ni. Yo ma gi maro tic kwede pi yango ni ikom coo tiyo ki yo ma two dong pe ibidwogo iyunge yango ne. Kwan ma nok me neno ayanga mu time ikom mon ma dok ilok kom two man ma mako man otime ento wa nwongo ni yo ma two man ma mako man otime ento wa nwongo ni yo ma yanga in coo ni wa nwongo ni perom ikom mon. Ikwan eni wa bi timo lapore ki wa neno ladwogi ne ka wa nwongo ni yo acel ca ni romo tici ikom mon kidire magi yubu pi. Wa bi poro gore enika romotic ikom mon.

Yoo me kwedo. Wa bi coyo mon 440 matye kitwo ni (hernia)kabedo ma patpat ilobo Uganda. Lutwo ma ikin jo wa coyo gini wa bi tic ikomgi ki yo manyen ikom two man. Jo mukene gi tic ikomgi ki yo ma pat magi ilwonyo ni Lichtenstein technique. Pe ibi ngeo yo ango magi tye katic kwede. Abara man daktal ma yango dano aye bi timo ne ki yat man mape iwinyo arem kagi tye ka yangi. Abara man bino tero dakika ikin 0 onyoo 120 ikwede cawa aromo aryo. Ki peko no oyaa ikom bar man kun latwo pe twero tunu otyat mo oyotwot pi neno daktal wa bi dwoki transport bang latwo. Teloki

Number pa latwo

ni ibi ngeyo komi ka gitye kabari ento pe iwiyo arem.Ingee abara ma inino no gicwali gang kaa arem pe ibiwot labongo ayella kagi nwongo ni itye kawinyo maber

mwaka acel daktal madit gi lwongi karoto ka itye maber nyoo pwodi ingee ayango man. Jo ama obin obedo kwan bino adwogi mere.

Jami ma ber ma wa nwongo ikom eni: Ibi nongo kony ma ayanga me nono. Cente me woti yi bi dwoko alip pyeraryo, 20,000. Kaa ulokaloka mo maber peke iwang reti,wabi neno yo mukene me cango twoman. Wabi lubu kor two man ingee cabit aryo, mwaka acel nyo mwaka adek wek wa nen kit me tic itwo ni ikare ne kikome.

Jami ma rac ma kwan eni mi ni wa ikom ayangomai .

Yange ne weng tye ki peko ma noknok ma romo bino. Lutwo mukene winyo arem ingee yango man mukene gi manok gi bi winyo arem ma pe tum. Iromo nwongo two ma nyen kare magi tye ka tic ikomi (yangi). Wa bi dwoko two ma ngole ikare me yangi piny. Dul daktari man bi nwongo yo ango me dwoko peko me nongo two mukene ni piny. Lutwo mukene two ni bi dwogo cen ikomgi(hernia). Wa bi dwoko latwo man ka yango ne ka two ni odwogo.

Ngat mu yee timo labongo dic: Yee ni tim gini man tye labongo dic. Ka iye winyo ningoli ma iniang ikom gin ma daktar owacini,obi miyi karatac eni weki iket cingi kan wek lanyut ni iye ceto ipala. Ka ityeko keto cingi kan abi penyi peny ma noknok ma lubu yot komi tutwale mukwako two ma kwako ikin emi ni. Kacel ma bino moko nino mo ma myero idwogi ot yat macok kwedi pi dwogo ibar. Gibi timo bar ne diki maca.

Number pa latwo

Daktari obi rwate kwedi ingee cabit aryo ingee bari me roto kita bar ni tye ka cang kwede. Ingee mwaka acel ki dok ingee mwaka adek.

Imung kwede mutini. Lok me yot komi ni imi owa eni ni wa bi gwoko bibedo imung ma pat kijo mubino ka kwedo tyen two eni ni. Lok komi ducu eni ni wabi gwoko maber wek pe onyute ka mukene ma pat ki bot jo ma tye ka kwedo. Ka itye ki peny mo ma kwako lok kom kwanene ni penya kwede, itwero go na cim pilapeny mukende (nying namba pa cin ne 0774 287 185). Obedo miti ni me bedo ikwan man dok dang itye agonya me weko ne, labongo peko moro keken icawa moro keken.

Dic mo peke me winyo pwonyere ene ni,ka idegi, iromo weko ne,gi mo ma rac peke lubu twero pa luwiny pwony eni ni onyo koko ma lubu pwony eneni obu tero atir bang Ms Harriet Chemusto (namba cim ne ene 0392 174 236 won kom madit me Mildmay Uganda Research and Ethics Committee. Med ki mano latwo room go cim bot ladwong kom(Research and Ethics Committee-RES) pi lok ma lube ki twero ki berbedo pa latwo.

Akwai ma pwod pa wa mede kii nyamo lok ene ni Amito lego twero ni lyee ni myero umede kwede ki pwonye ene ni?

Gigonya pwony eneni Maber adada dok anyang kwede bere ki pire ne? Aniang matut ikom pwony eni ni dok gi waca jami ne ama mite. Lapeny weng duc gigamo mu yomo yia. Aye dong me bedo ipwonye eni.

Number pa latwo

Nying Latwo………………………………………

Cing pa latwo ……………………………………..

Ninodwe…………………………

Pe ngeo coc,twon cing.

Nying caden pa latwo (ka latwo pe ngeyo coc)…………………………………..

Cing pa caden ……………………………………..

Ninodwe…………………………

Nying ngat ma tye ka gamo nyo coyo, twero ibot latwo.

Cing dano ma tye ka gamo twero ibot latwo…………………………………..

Ninodwe………………………………………

Appendix 2. **Number pa latwo**

Namba aryo:

Kara tac ma kwako kom Lat

Ngat mu oponyo obedo……………………………

Nino dwe ki kabedo ne……………………………

A. Jamima kwako kom latwo………………………

1. Nying pa latwo………………………….............................
2. Distric pa la two………………………………………………….
3. Nying caro palatwo……………………………………………….
4. Namba cini ne ………………………………………………….
5. Namba cini ne ………………………………………………….
6. Namba cini ne …………………………………………………..
7. Nying Chairman…………………………………………………..
8. Namba cini pa LC1………………………………………………….
9. Tic ne………………………………………………………………..
10. Ka tic ne…………………………………………………………

Number pa latwo

11.Namba cini okene obedo…………………………………………

……………………………………………………………………….

……………………………………………………………………….

……………………………………………………………………….

12.Mwaka pa la two……………………………………

13.Imato taa?..

14.Yi tye?..

a.Ka tye ,ter bang daktari

15.Onyo itye ki two ma pe cang?

1.Tye 2. Peke.

16. Ka tye , two mene ni?

17. Iti kwede two cukari?

1. Tye 2. Peke 3. Pe angeyo.

18. Iti kwede two cwiny?

1. Tye 2.Peke 3. Pa angeyo.

19. Iti ki two mo ma mako oboo?

1. Tye 2. Peke 3. Pa angeyo.

20. Itii ki peko ma ka inwongo awano remo cwer majuko ne teki?

1 Tye 2 . Peke 3. Pa angeyo.

21. Itii ki two mo makobo oyotyot ikomi kombedi?

Number pa latwo

1 Tye 2. Peke . 3 Pe angeyo.

22. Ka tye mene……………………………………….

1. Hep B, 2 Hep C, 3 HIV 4 Gola Wio, 5 Mukene………..

23. Itii ki yat mo ma mwonyo juijui?

1. Tye 2. Peke 3. Mukene……………………………..

24. Ka tye, yat mene

1…………… 2………… 3…………4……………

25. Dok ki bari ki pwodi? ……………………………

26. Ka kibari,obedo awene?

1. Mwaka acel 2. Mwaka acel angee 3. Pe angeyo 4. Mukene. ………

27. Ka ki bari,two mene mu ki bari kwede ni?

1. Ikin ema 2 Pe angeyo 3. Mukene

Number pa latwo

28. Ka ki bari, obari kwene?

1. Ot yati Madit, 2 . Ot yat matidi, 3. Otyat pa dano mo.

29. Itii ki akwotakwota mo ma okwot ikini emi?

1. Peke 2. Tung cem 3. Tung cam 4. Nyongo weng, 5. Mukene…………..

30. Iwinyo arem mo ikin emi?

1.Pe

2. Arem tye ma noknok aromo kanyo ne.

3. Arem tye mawinyo jwii ento aromo kanyo atiyo kwede.

4. Arem tye matek pe aromo kanye ne dok balo tica weng pe aromo tiyo ne.

6. Arem tye matek ma pearomo nino pe aromo kanyo ne.

7. Arem tye matek mada ma pa twero timo gi mo keken myero nea ceti iot yat

31. Ibedo ka winyo arem ma rem arom mene ikin emi?

1. Dwe aromo 0-6, 2. Ma kato dwe abicel (6). 3. Pe angeyo.

32. Ibedo minyo arem ma rem dwog ikin emi?

1. Ongo , 2. Pe 3. Pe engeyo.

33. Itii ki, lok mukene onyongo mo me medone?

………………………………………………………………………………

Appendix 6.

Number pa latwo

Namba abiro

Karatac me lubukor latwo ingee mwaka adek:

Ngat mupong obedo…………………………………

Nino dwe ki kabedo ne………………………………

Jami ma kwako kom latwo.

1. Nying palatwo…………………………………………..
2. District Palatwo…………………………………………
3. Nying caro palatwo…………………………………….
4. Namba cini ne…………………………...........................
5. Namba cini ne ……………………………………………
6. Namba cini ne ……………………………………………
7. Nying chairman……………………...........................
8. Namba cini pa LC1………………………………………
9. Tic ne……………………………………........................
10. Ka tic ne (katye)…………………………………………..
11. Jami mukene obedo……………………………………….

……………………………………………………………..

……………………………………………………………..

Number pa latwo

12. Ono kit peko mo keken marac ingee bar ma baroyin ma kwot ikin emi ni?

1=Eh, 2= ku, 3=mukene.

13. Ka inongo peko marac , kit mene ni?

1. Arem, tye matek mukato ma mito lakwe arem mukene.
2. Cwer ka remo,del koma oloke kakama ibaro ni.
3. Cwer ka remo,tele pa del kom, pe loke ka del kom keken.
4. Two mukene , ma mito yat matek.
5. Two ma mite mi ni wang bur ki yap.
6. Peko ma mite ni kiket lupira lac.
7. Remo ma make yi oboo onyo tye.
8. Aola pneumonia.
9. Two Adunu
10. Awogo atwo akwota ene(hernia)
11. Mukene.

14. Ka onongo pekomarac tye, obedo matek marom kwene?

1.Pe tek, 2= Tek, 3=Tek Madit, 4=Tek Madit adada.Ma mito kwanyo kwo na.

15. Iyeng adugi ma bar ni en?

1= Eh, 2= ku, 3= Mukene.

16.Iceto iot yat ingee bar maki timo ni?

1= Eh, 2= ku

Number pa latwo

17. Iye ni laremi mo nyo laremi mo abar pi hernia ki yat ma dingo areme? ☐

1= Eh, 2= ku, 3= Mukene.

18. Itamo ni myero wa mede ki baro dano kitwo akwota calo two mukene ene ni? ☐

1= Eh, 2= ku, 3= mukene

19. Lok mukene kwede tam muken. ☐

……………………………………………………………………………………………
……………………………………………………………………………………………
……………………………………………………………………………………………
……………………………………………………………………………………………
…………………

Appendix 7.

Number pa latwo

Namba abicel.

Karatac me lubu kor latwo mwaka adek….

Ngat mupongo abedo…………………………….

Nino dwe ki kabedo ne……………………………

Jami ma kwako kom latwo.

1. Nying palatwo…………………………………………………
2. District Palatwo……………………………………….............
3. Nying caro palatwo……………………………………............
4. Namba am ne………………………....................................
5. Namba am ne…………………………………………………
6. Namba am ne………………………………………………..
7. Nying chairman……………………….................................
8. Namba cini pa LC1……………………………………………
9. Tic ne………………………………….................................
10. Ka tic ne(katye)………………………………………………..
11. Jami mukene obedo……………………………………………

Number pa latwo

Lapeny eni tye ikom gurem ma itye kwede kambedi onyo matye ikini emi icabit mukato

12. Wac ngo ma inwongo marac ikom bar mutime ikin emi ni

    1. Arem pe
    2. Arem tye ento pe tutwal
    3. Arem tye ento gengo tica jwi
    4. Arem tye ento gengo tica jwi
    5. Arem tye ento gengo tica jwi jwi weng
    6. Arem tye ento gengo nino na iwor
    7. Arem tye ma mito kong iot yat

Ka latwo ogamo ni arem pe ilapeny namba 12, mede ki lapeny namba 13. Kwede namba 17 naka 20.

13. Ka igamo ni larem obedo ila peny namba apar (10), wek wi kupo gwere marem ma obari ene ni ikin emi orweny.

    1. Ingee dwe acel larem orweny
    2. Ingee dwe acel ooh kwede idwe adek
    3. Ingee dwe angwen ooh kwede idwe abicel
    4. Ingeee dwe abiro ohh kwede idwe apar aryo
    5. Pwodi orweny macok coki.

Number pa latwo

Ka latwo onwongo arem icabit mukato ni, gam lapeny namba 14 kede 15.

14. Arem binno nini icabit mukato ni?

1. Icel cabit acel.
2. Iryo onyo ibic icabit acel
3. Nino weng
4. Nino weng nyo idiwor weng
5. Abedo ki larem icabit weng, idiwor wa idiceng.

15. Onyongo inwogo yat ma dingo arem iyi larem ma tye ikim emi ni icabit mukato?

1. Ku, 2= Eh, 3= Mukene

16. Marom kwe ma larem eni ni ikin emi egengo in tiyo tic ni idwe aryo mukato?

1. Abedo ka tiyo tic na maber macalo kare mukato
2. Larem ni ogengo an ki tiyo tic na macato kare mukato.
3. Pi nino maromo acel ooh kwede iyi abiro idwe aryo mukato.
4. Larem ni ogengo an ki tiyo tic na macalo kere mukato
5. Pi nino maromo acel ooh kwede iyi angwen idwe aryo mukato.
6. Larem ni ogengo an ki tiyo tic na macalo kare mukato pi dwe aryo mukato ni weng.

Number pa latwo

17.Iwinyo arem nini tin ki peko mukene kan ma ibaro ikin emi ka oparo kwede ma pwod pe ki baro? Romaroma,Kato onyo nonak?

1=Nonok, 2= Romaroma, 3= Dwong mukato.

18.Icang maber iyi bar ma baro ikin emi ni?

1= Eh, 2 Ku, 3=Mukene.

19.Kip e, Peko mene ma dong itye kwede.

1= Larem, 2= Peko me wot, 3= Mukene

20.Iyeng ki ladwogi me abara ama ki baro ki ikin emi ni?

1= Eh, 2=Ku

21.Doki ki bari anaka ikin emi onyongo iyii anaka labara me kini eni ni ……………….. iot yat imwaka 20….

1=Eh, 2= Ku , 3= Mukene.

Appendix 8. **Number pa latwo**

Namba aboro.

- Karatc me ladwogi bar ma ki bar ki lutwo.

- Lanyut mago ma bi bedo tye kwede jami ma myero itim kwede pe itim ingee bari.

- Inge bari ibi winyo larem marok, yat ibrufen kwede panado twero tic mabeer pi kweyo larem eni icawa weng. Pe myero iti ki aspirin onyongo diclofenac me pwod pe ki bari pien gi kero kero me cwer pa remo.

- Pi larem, pe ubi bedo ki ker me timo tic maber calo kare mukato pi kare maromo 10-14 ingee bar eni. Ento pire tek me yin wot onyo timo exercise.

- Cwer remo ma nok idel kom bedo tye, gi twero loko colour me wang bar ni kede dang itong man. Man pe rac dang rwenyo ako ingee nino 30.

- Wang bar ni eni obi bedo matek iyi dwe maromo acel ingee bari ento obi doko mayom ingee kare manok.

- Kwe kom ma cok ki kan ma obaro bedo tye ento pe obedo gin marac pien obi rwenyo woko.

- Wa bi gongo wuci iwang ret ma wa baro ni ingee cabit aryo. Inino no cutcut daktar madit obi neng neno ka wang bar tye ma ber wek ki geng gimo marac ma mito bino con.
- Jami mo marac mamito kony pa daktal.
- Two ma mukene ma nen iwang bur magi bari kwede ni: wang ret onyo bur ono wang pala oromo kwot madit kwede larem madit, licawa mukene tut pelo iwange ni.

- Akwotakwota: Remo ma bedo cwer ma nok idel kom twero kelo akwootkom iwang bar ma ki bari kwede ni.

- Ka inongo jami marac eno ni ingee bar man, wa kwai me goyo cini bang daktali madit Dr. Alphonsus Matovu, inamba cini ne 0774 287 185 wek en niang peko eni tye planning pi roti doki ka twere

Number pa latwo

Plan me rota lutwo ingee bar man ingee nino 14 bi bedo inino dwe……………….. cawa …………… kwene ……………………… daktal mene……………………..

Addendum 3: Luganda translation.

Enamba y'omulwadde.

Appendix 1.

Consent form.

Entandikwa.

Amanya gange nze …………………………. Nga ndi omu kwabo abali mu kunonyereza okuyitibwa okugerageranya okulongoosa hania mu bakyala nga bakusaze ne batekamu akatimba nga bwe kikolebwa kati ne ngeri empya era nga bakusaze ne batekamu akatimba naye nga bakyusizaamu engeri gye bakatemu. Okunonyereza kuno kumaze okukasibwa akakiiko mu kitongole kya Mildmay Uganda akalina olukusa okwekebejja ebyo kunonyereza mu Uganda.

Ekigendererwa. Abantu bangi balina obulwadde bwa hania. Mu basajja abakulu, mu buvanjuba bwa Uganda kyazulibwa nti abasajja kumi ku buli kikumi balina obulwadde bwa hania. Mu basajja, enkola emu eyokulongoosa hania, ekozesa akatimba okugumya mu bitundu byo mu mawago okukendeeza emikisa gya hania okudda. Emisomo mitono nnyo egikoleddwa okunonyereza ku kulongoosa hania z'omumawago mu bakyala okuzuula enkola esinga obulungi kuba kimanyiddwa nti enkola ekozesa akatimba mu basajja okulongoosa hania tekola bulungi mu bakyala. Mu musomo guno tugenda kunonyereza oba akatimba akakozesebwa okulongoosa hania kasobola okukola mu bakyala nga tukyusizaamu engeri gyetukatekamu.

E nnamba yo mulwadde.

Emitendera gyokunonyereza. Tujja kusaba abakyala 440 abalina hania mu mawago okuva mu bitundu bya Uganda ebye njawulo okwetaba mu musomo guno. Abakyala abamu bajja kulongosebwa mu ngeri yemu nga bwe tukola mu basajja nga tukozesa akatimba, ate abalala balongosebwe nga tukozesa engeri empya gye tugezesa mu bakyala.Munkola eno egenda

okugesebwa, akatimba tukasala netubika emiwatwa gyonna hania gyasobola okuyitamu mu bakyala. Oyo anateba mu musomo guno tajja kumanya ngeri ki gya longosedwamu.

Okulongoosa kujja kolebwa abasawo abakugu mu kulongoosa. Omusawo agya kusanyalazaako awo awali hania wokka. Tojja kuwulira bulumi era ogya kuba nga otegeera mu kiseera ekyo kukulongoosa. Okulongoosa kujja kumala wakati w’edakiika nkaaga ne kikumi mu abiri oba wakati we ssawa emu ne bbiri.Okulongoosa nga kuwedde, ogya kutambula wekka, abasawo bwe banalaba nga osobola okuddayo ewaka, bajja kusiibula ogende ewaka. Oluvanyuma lwa sabiiti bbiri, n’oluvanyuma lwomwaka, abasawo bajja kuddamu okukubera balabe engeri gyowonyeemu. Oja kutegezebwa engeri okunonyereza kuno nga bwe kutambula wamu nebyo ebizuuliddwa.

Omugaso ogunaava mu kwetaba mu musomo guno.

Hania egya kulongosebwa, ogya kuwebwa entambula ya sh.20,000 eza Uganda buli lwonojja mu ddwaliro. Ekiteeza nti omulundi ogusooka lwonojja, omulundi ogwo

E nnamba yo mulw adde

kubiri nga wayise week biiri ng’omaze okulongosebwa, okukeberebwa oluvanyuma lwo mwaka era ne ku myaka esatu ng’okulongoosa kuwedde.. Singa waberawo obuzibu bwonna, abasawo baffe bajja kubukolako.

Bwonooba ofunye obuzibu nga wetaaga okujja mu dwaliro mangu naye nga tosobola, tujja kukuyambako okutuuka mangu mu dwaliro. Bwonooba tofunye buzibu bwa mangu,oja kukeberebwa oluvanyuma lwa sabiiti bbiri, omwaka ne myaka esatu abasawo okwongera okulaba engeri gy’owonyemu.

Obuzibu obuyinza okuva mu kwetaba mu musomo guno.

Okulongoosa kwonna kubeera n’obuzibu. Waberawo obulumi, ate abamu obulumi bumala ebbanga ddene. Ekiwundu kisobola okutana. Obuzibu bwona bujja kukendezebwa nga tukozesa enkola ezomutindo mu kulongoosa okwewala obuzibu obuyinza okuberawo. Mu bamu hania esobola okudda, era bwe nekomawo tujja kuddamu tukulongoose hania.

Okwetaba mu musomo guno kwa kyeyagalire.

Okwetaba mu musomo guno kwa kyeyagalire. Bwonokiriza okwetaba musomo guno , tujja kusaba okutuwa ekinkumu oba okuteeka omukono ku mpapula eziraga okukiriza kwo okwetaba mu musomo guno. Ekyo nga kiwedde, nja kukubuuza ebibuuzo ebikwata ku bulamu bwo nebikwata ku hania. Nze nawe tujja kukanya olunaku okujja ku dwaliro

wetunateeka teeka okulongoosa kuno. Okulongoosa kujja kukolebwa ku lunaku oluddako ng'omaze okukeberebwa singa tewabawo bitataganyizza ntekateka.

E nnamba y'omulwadde

Omusawo waffe aja kusisinkana oluvanyuma lwa week biri ng'okulongoosa kuwedde , oluvayuma lw'omwaka wamu n'oluvanyuma lw'emyaka esatu.

Ekyama no bwebange.

Ebyo byonna byonotubulira bijja kumiibwa butiribiri, era bijja kumanyibwa abo bokka abali mu kunonyereza kuno. Ojakuwebwa omuwendo ogunawandiikibwa ku mpapula zo, amanya go tegajja kulagibwa ku mpapula , nemukugatta ebivudde mu byetukungayizza wamu ne kumubiwandikako mu bitabo ebye njawulo, amanyago tegajja kulagibwa. Bwoba olina kyo buuza mbuuza oba oyinza okubuuza omunonyereza omukulu(Amanyage ne number ye eye simu bibino. Dr. Matovu Alphonsus, 0774 287185). Kiva mu kusalawo kwo okwetaba mu kunonyereza kuno era oli wa ddembe okuvaamu esawa yonna , tewali buzibu bwonna bujja kukutuukako.

Ebibuuzo ebikwata ku ddembe lyo eryo bwabange okwetaba mu musomo guno,bisobola okubuuzibwa Ms Harriet Chemusto ku simu number 0392 174236 sentebe wakakiiko akakola ku byo kunonyereza mu Mildmay Uganda Research and Ethics Committee. Era Oli wa ddembe okutukirira omukulembeze wakakiiko akakasizza okunonyereza kuno (Mildmay

Uganda Research and Ethics Committee) okumanya ebikwata ku ddembe lyo wamu ne ndabirira yo mu bbanga elyo kunonyereza kuno.

Okukirizza. Nga tetunaba kweyongerayo na bibuuzo, nkusaba okirize okwetaba mu kunonyereza kuno. Okirizza okwetaba mu kunonyereza kuno?

E nnamba y'omulwadde

Nyinyoleddwa mu bujjuvu byonna ebikwata ku kunonyereza kuno era ntegeera ebigenderera byakwo. Ntegedde mu bujjuvu ebyetagisa okwetaba mu musomo guno. Ebubuuzo byange byonna biddidwamu bulungi. N'olwekyo, nsazeewo okwetaba mu kunonyereza kuno.

Erinya lye yetablye mu kunonyereza. ……………………………………..

Omukono gwoyo eyetabye mukunonyereza……………………………………..

Enaku z'omwezi…………………………………………………………….

Ekinkumu kya yetabye mu kunonyereza……………………………

Erinya ly'omuyima singa eyetabye mu kunonyereza tasobola kuwandiika

Omukono gw'omuyima……………………………..

Ennaku z'omwezi…………………………………..

Erinya ly’oyo abuuza ebibuuzo………………………………………………

Omukono gwoyo abuuza ebibuuzo…………………………………………….

Ennaku z’omwezi. ……………………………………………………………..

Appendix 3. Ebikwata ku bulamu bwo.

E namba y’omulwadde

Ajjuzaamu---

Ennaku z’omwezi--

A. Endagiriro y’omulwadde.
   1. Amanya……………………………………………………
   2. Erinya lya disitulikiti gyobeera ……………………………...
   3. Erinya lye kyalo gyobeera…………………………………….
   4. E number yo eye Simu 1………………………………………
   5. E namba yo eye simu 2………………………………………..
   6. E namba yo eye simu 3………………………………………..
   7. Erinnya lya Chairman we kyalo……………………………….
   8. E namba ye simu eya Chairman we kyalo. ……………………
   9. Okola mulimo ki?...
   10. Okolela wa……………………………………………..
   11. Olina engeri endala gyoyinza okutukirirwamu?

……………………………………………………………………..

……………………………………………………………………..

E namba yo mulwadde

12. Olina emyaka emeka?...

13. Onya Sigara?...

14. Olina olubuto?..

1. Ye. 2. Nedda. 3. Kisoboka. 4. Ebirala byonna.

15. Olina obulwadde obuluddewo?

1. Ye 2. Nedda.

16. Bwoba obulina, olina bulwadde ki?

………………………………………………………………………………..

……………………………………………………………………………….

17. Oli mulwadde wa sukaali?

1. Ye 2. Nedda. 3. Simanyi 4. Ebirala byonna.

18. Olina obulwadde bwo mutima?

1. Ye 2. Nedda 3. Simanyi 4. Ebirala byonna.

19. Olina obulwadde bwonna obukutawanya mu mawugwe?

1. Ye. 2.Nedda. 3. Simanyi. 4. Ebirala byonna.

E nnamba y'omulwadde

20. Bwe wabawo ekikusaze, ovaamu nnyo omusayi nga tegulekerawo

1. Ye. 2. Nedda. 3. Simanyi.

21. Olina obulwadde ng'obwa siriimu, hepatitis oba TB?

1. Ye. 2.Nedda. 3. Simanyi.

22. Oba ye, bulwadde ki?

1. Hepatitis B, 2. Hepatitis C, 3. Siriimu, 4. TB, 5 Ebirala byonna.

23. Olina edaggala lyomira buli lunaku?

1. Ye 2. Nedda. 3. Simanyi .

24. Oba ye, ddagala ki?

1……………………………………………………………

2.……………………………………………………………

3…………………………………………………………….

4…………………………………………………………….

25. Walongoosebwako?

1. Ye. 2. Nedda. 3. Simanyi 4. Ebirala byonna(Nyonyola).

26. Oba ye, walongoosebwa ddi?

1. Mu mwezi nga gumu emabega. 2. Omwezi gumu guyiseewo. 3. Simanyi. 4. Ebirala byonna(Nyonyola)…………………………………

27. Oba ye, bakulongoosa ki?

1. Hania mu mawago. 2. Sim
2. anyi. 3. Ebirala byonna.

28. Bwe kiba yi, bakulongoseza wa?

E nnamba yo mulwadde

1. Mu ddwaliro 2. Edwaliro eddala elya government 3. Mu Clinic eyo bwananyini 4. Simanyi 5. Ewalala wonna(Nyonyola)………………..

29. Ofuna okuzimba mu mawago oluyi lumu oba gombi?

1. Nedda. 2. Oludda olwa ddyo 3. Oludda olwa kono 4. Ebira byonna(Nyonyola).

30. Owulira obulumi mu limu oba mu mawago gombi?

1. Siwulira bulumi
2. Obulumi webuli naye sibufaako.
   Ku ddyo Ku kkono
3. Obulumi webuli, mbuwulira naye tebunemesa mirimu gyange gya bulijjo.
   Ku ddyo Ku kkono
4. Obulimi webuli, sisobola kubuleka,bunemesa emirimu gyange eja bulijjo.
   Ku ddyo Ku kkono
5. Obulumi mbuwulira, sisibola kubuleka era bunemesa okukola emirimu egisinga.
   Ku ddyo Ku kkono
6. Obulumu webuli , sisobola kubuleka era bunetaagisa okuwumula.
   Ku ddyo Ku kkono
7. Obulumi webuli, sisobola kubuleka, bwetaaga okufuna obujanjabi obwa mangu.
   Ku ddyo Ku kkono

31. Obulumi mu mawago bumaze banga ki?

0 Okutuuka ku myezi 6,   2. Busukka mu myezi mukaaga, 3. Simanyi.

E nnamba y'omulwadde.

32. Wafunyeko obulumi obwa manyi mu mawago?

Ye. 2. Nedda. 3. Simanyi.

33.   Waliwo ekirala kye wandyagadde okwogerako?

……………………………………………………………………………………….

Appendix 6.

Okukebera ayetabye mu musomo oluvanyuma lwa sabbiiti bbiri.

E namba y'omulwadde

Ajjuzzaamu---

Ennaku z'omwezi---

B. Endagiriro y'omulwadde.

1. Amanya ………………………………………………………..
2. Erinya lya distulikiti gyobeera……………………………………….
3. Erinya lye kyalo gyobeera……………………………………………
4. E namba yo eye simu 1………………………………………………
5. E namba yo eye simu 2……………………………………………..
6. E namba yo eye simu 3………………………………………………
7. Erinya lya chairman we kyalo……………………………………….
8. E namba ye simu eya chairman we kyalo…………………………..

E nnamba y'omulwadde

9. Okola mulimu ki?...

10. Okolera wa...

11. Olina engeri endala gyo yinza okutukirirwamu.................................
    ………………………………………………………………………
    ………………………………………………………………………

Ebibuuzo ebikwata ku mbeera ng'omaze okulongosebwa.

12. Ofunyemu obuzibu bwona ng'omaze okulongosebwa?
    1 = Ye. 2= Nedda.
    3= Abirala.
    Nyonyola……………………………………………………………….

13. Bwoba nga wafunye obuzibu, wafunye buzibu ki?
    1. Obulumi obwa manyi enyo nga bwetagiisa amakerenda agamaanyi enyo, okwongera kugebakuwadde oba okugakyusizza ddala.
    2. Okuvaamu omusaayi ,wamu nolususu lwe kiwundu okukyusa langi.
    3. Okuvamu omusayi, okuzimba n'olususu okwereega si kukyusa langi kyoka.
    4. Okutana ng'ekiwundu kyetaaga eddagala.
    5. Okutana ng'ekiwundu kyetaaga okuddamu okutagululwa.

6. Okulemererwa okufuyiisa nga kyetagisa okukuyisaamu akapiira.
7. Omusaayi okwekwata obutole mu kugulu oba mu mawugwe.
8. Lubyamira

E nnamba yo mulwadde

9. Omutima okusanyalala mu bitundu ebimu
10. Hania okuddamu
11. Ebirala……………………………………………………………………
……………………………………………………………………..

14. Bwe waba nga waliwo obuzibu, bwali bwe nkana ki?

1=Sibwamanyi nnyo, 2= Obuzibu buli wakati awo. 3= Obuzibu bwa manyi

4=Buyinza okusanyawo obulamu. ……………………………………..

15. Oli musanyufu ne bivuddemu mu kulongoosebwa?

1=Ye, 2= Nedda, 3= Ebirala. …………………………………………

16. Wafunyeko obujanjabi bwa hania oba ekiwundu ng’omaze okulongosebwa?

1=Yes, 2= Nedda…………………………………………………………

17. Osobola okusikiriza mukwanogwo oba ow’oluganda okulongosebwa hania nga basanyalazizza hania yekka?

1= Ye, 2= Nedda, 3=Ebirala………………………………………………

18. Olowooza nti okulongoosa hania kugende mu maaso nga abalwaddee bajja kwolwo, ne balongosebwa ate ne baddayo eka?

1= Ye, 2= Nedda………………………………………………………………..

19. Waliwo ekirala kyonna kye wandyagadde okutubulira?

…………………………………………………………………………

Appendix 7.

Okukeberebwa oluvanyuma lw'omwaka.

E namba yo mulwadde

Ajjuzzaamu---

Ennaku z'omwezi--

C. Endagiriro y'omulwadde.

1. Amanya……………………………………………………………….
2. Erinya lya distulikiti gyobeera ……………………………………..
3. Erinya lye kyalo gyobeera………………………………………….
4. E namba yo eye simu 1………………………………………………
5. E namba yo eye simu 2…………………………………………….
6. E namba o eye simu 3……………………………………………….
7. Erinya lya chairman we kyalo………………………………………
8. E namba ye simu eya chairman we kyalo………………………….
9. Okola mulimu ki?...
10. Okolera wa?...
11. Olina engeri endala gyo yinza okutukirirwamu…………………….

……………………………………………………………………....

…………………………………………………………………...

Enamba yo mulwadde

Ebibuuzo ebiddako bikwata ku bulumi bwolina kati oba bwobadde nabwo mu week eziyise.

12. Tebeleza obulumi obusinga amanyi bwofunye mu mawago week eyise.

1. Sirina bulumi.
2. Obulumi webuli naye sibufaako.
3. Obulumi webuli, mbuwulira naye tebunemesa mirimu gyange gya bulijjo.
4. Obulimi webuli, sisobola kubuleka,bunemesa emirimu gyange eja bulijjo
5. Obulumi mbuwulira, sisibola kubuleka era bunemesa okukola emirimu egisinga.
6. Obulumu webuli , sisobola kubuleka era bunetaagisa okuwumula.
7. Obulumi webuli, sisobola kubuleka, bwetaaga okufuna obujanjabi obwa mangu.

If the patient answered no pain in question 12, please continue with question 13 and then questions 17 -20.

13. Bwoba wazeemu " Siwulira bulumi" ku kibuuzo namba 10 waggulu, gezaako ojukire obulumi ku ludda olwa longoosebwa lwe bwagenda ng'omaze okulongosebwa.

1. Obulumi bwagenda mu mwezi gumu.
2. Obulumi bwagenda wakati womwezi gumu ne satu.
3. Obulumi bwagenda wakati we myezi ena n'omukaaga
4. Obulumi bwagenda wakati we myezi musanvu ne kumi nebiri.
5. Obulumi bugenze emabega ko awo.

E namba yo mulwadde

If the patient has felt pain in the operated groin during the past week, please answer question 14 and 15.

14. Obulumi ku ludda olwalongosebwa, obadde obuwulira otya?

   1. Omulundi gumu buli week
   2. Emirundi ebiri okutuuka ku etaano
   3. Buli lunaku
   4. Buli lunaku wamu ne kiro
   5. Mbadde n’obulumi week yonna , emisana ne kiro.

15. Wamizeeko amakerenda g’obulumi ng’omaze okulongoosebwa week eyiseewo.

   1= Nedda, 2= Ye, 3= Ebirala.

16.Obulumi bwolina mu mawago butuuse wa mu kukulemesa emirimu gyo oba okukola emirimu gyo buli lunaku mu myezi ebiri?

Njagala kugeragerenya obunene bwo bulumi bwolina nobusobozi okukola emirimu gyo buli lunaku.

1. Mbadde nkola emirimu gyange egya buli lunaku nga buli jjo.
2. Obulumi bwa nemesa okukola emirimu gyange egya buli lunaku wakati w’olunaku lumu n’omusanvu mu myezi ebiri egiyise.

3.Obulumi bwa nemesa okukola emirimu gyange egya bulijjo wakati wa week emu ne nnya mu myezi ebiri egiyise.

E namba yo mulwadde

4.Obuli bwanemesa okukola okumala emyezi ebiri gyonna egiyiseewo.

17.Obulimi bwolina kati obugerageranya otya ne mbeera ku ludda olwalongoseddwa nga tonaba kulongoosebwa? Kye kimu, bukendedddde? Oba bwe yongedde.

1= Bukendedde, 2= Kye kimu, 3= Bwe yongedde, 4= Ebirala.

18. Owonye bulungi ku ludda olwa longoseebwa?

1=Ye, 2= Nedda, 3= Ebirala.

19.Oba nedda, buzibu ki bwolina kati?

1=Obulumi, 2= Sisobola kutambula bulungi, 3= Nina obusanyalavu.

20.Oli musanyufu ne bivudde mu kulongoosa hania?

1= Ye, 2= Nedda.

21. Wafunyeko okulongosebwa hania oba okulongosebwa ku lubuto ng’omaze okulongosebwa hania? ………………………. Eddwaliro , ennaku z’omwezi.

1= Ye, 2= Nedda, 3= Ebirala.

Appendix 8.

Obubaka obuwebwa omulwadde ng'amaze okulongosebwa.

Enamba yo mulwadde.

Ebisuubirwa ne byetaagisa okukola ng'omaze okulongosebwa.

Obunero bwebyo ebisuubira nebinakolebwa nga okulongoosa kuwedde.

- Ng'omaze okulongosebwa, oja kuwuliramu obulumi obutonotono. Panado aja kuba amala okukendeeza obulumi buno ebiseera ebisinga. Totekeddwa kukozesa aspirin oba dikulofenaka kuba byongera okuvaamu omusaayi.
- Olwobulumi , oyinza obutasobola kukola murimu gyo egya bulijjo mu week 10 ku 14 ezisooka nga wakalongoseebwa. Wabula , kyamugaso nnyo okukozesa omubiri gwo , ng'okola exercise,ng'okutambulako nga bulijjo.
- Oyinza okuvaamu omusaayi omutonotono. Ekiwundu kiyinza okukyusa langi oba okukyusa langi ku lusuusu. Kino tekirina buzibu kigyakugwawo mangu.
- Enkovu egya kuba nene mu mwezi ogusooka nga wakalongoseebwa. Nga wayise akiseera ,enkovu egya kugenda.
- Okuwulira obusanyalavu ku nkovu kitera okuberawo naye tekirina bulabe
- Wuzi zijja kujibwamu nga wayise enaku kumi nanya. Omusawo agya kukukebera okusobola okuzuula obuzibu ku kiwundu amangu.

Obuzibu obutonotono obwetagisa omusawo.

Ennamba yo mulwadde

- Ekiwundu okutana. Ekiwundu kizimba, kiruma era amasira gavaamu.
- Omusaayi okwetugga. Omusayi guvaamu ne gwetugira mu kiwundu.
- Bwoba ofunye obuzibu bwonna obumenyeddwa wagulu oba obuzibu obulala bwona, tukuwa amagezi okukubira Dr. Alphonsus Matovu ku simu namba 0774 287 185 okwogera ku buzibu obwo era n’okufuna okuberebwa okwetaagisa.

Okukeberebwa nga wayise sabiiti biri kujja kuberawo nga …………………………

Esawa……………………….. ………………… Ekifo……………………….

Nga kukolebwa Dr………………………………………………………...

Addendum 4: Lugbara translation

Appendix 1 **Number azoba onitaa niri**

Fomu onitaa niri

Edozu. Ma aru ………………………………. ma esele aziba oninia onipigeriko eri bani hania olisori, ma esele cosuri oku marua ri andrale ripi ma eselea orule dide. Onita nderi committee eyo adani(Mildmay Uganda) nirise edeni.

Asisile. Haia agaleniri eri ao adepiri wudrikuru sini. Agupi Uganda ma woko etuni efuria diyi ma esele 10/100 ba esu adra eyi azo nde be iota okonisi . Onita diyi ma alea , ba nga esele coza geriko oku bani oku pima hania otuzuri vini geriko bai agupi pima hania otuzori esu.

Geriko onitari: Ba nga oku 440 hania ageleni beri esu Uganda ma kalatasi. Eyi ma esele diyi ba nga oli geriko drio bani okuyi olizurisi, azi diyi ba nga ali geriko bani agupi pima geriko otuzo okunirisi. Ba nga asiza nderi de sawa 1 kipere iri dipi me alea. Ba nga oku nde eyi so aro ba ecozu suza arizo kurisi vini bani ngazo mile be mbedo bai operesoni nde ederiarisi. Liza ma vutia minga ali mii lelerite, azii ko ecora, mi eco mvi akua cotira. Yumula olu azini eli alu ma vutia ba nga mi ozi azini dokitari ninga mi ne olika lita nderi ma efula ngoniari esuzu. Ba onita niri eringa eyo onita ndeniyi modria eyo oni .

Orodri onitaa ndeneri**:** Minga operesoni(lita)hania niri esu pirini sente kokoru. Minga silingi esu 20,000 ajiparu. Ba nga hania miniri asi aje kokoru. Minga vini silingi alivu kaliri ri esu saa dria bani ngazu mi lezu nezu sawa onitaa di vulesi. Sawa bani mi sizurisi,eli alu mavutia,vini eli na ma vutia. Eyo azini onzi ka nga eda mima rubatiara,ba nga ofu mii ra. Yamula 2 eli,vini 3 vutia,banga mi ne mini muzo azo azini opkoruni esuzi ku beni. Azini eyo ewaru ka osumideci. Mini ama azakomalezuni echozu cosu arajua mbele ku amanga nini nditria fee emozu raa.

Onzirikana onitaru, Liza operesoni woro eyi onzirikana be. Azoba eco suza esu operesoni vutia ra, azii ba werearu diyi eco suza onzirikanaruri esu va. Ba out diyi

Number azoba onitaa niri

esizu ra. Azoba werea diyi nga hania esu dika . Ama vini nga eima hania efupi dikari lii ra. Pazi duza misi ceneri. Minga ovu onita diyi ma aziru misi ceni. Eka olio vu onitaa diyi ma ba azi ru va,minga dri ti karatasi diyi ma dria mima aita ecezu. Da ma vutia , ma nga mi ozi zitasi mi ma alataa vini mima hania agaleniri ma dria. Ama tualu mibe odu operesoni edezuri otuu hospitali mi ma ageyia ogogoria. Ba nga mi li odu dasi coti. Dakitari ni nga mi ne dika yumula 2 nosiku alu mavutia ani eyo ewaru kami es bani azanikosu mbeleru coti

Eyo ziza: Eyo mii ece amai onita diyi edezari ba nga tani mba muke tezu ba oitaa nderi edba diyi ni eri esuzo ni adusi. Miru cani nga efu efuta ripotiru banis nga mu efuri ma ria ku . Mi ma eyo azi mini le oni onitaa nderi ma driani ka adre cii,ezi ma i. Di ma vutia mi eco eyo cini zi ma tia ra(ma ru telfoni nam 0774 287 185. Eri drile mgobo mini adrezu onita diyi ma baru,azini mi eco fu onitaa diri ma aleasriste sawa liria,vini eyo azini kokoru. Eri aki mini ovuzoo onitaa di ma aziru, azini mi eco oazu onitaa nderi ma azi ku sawa ciria.

Eyo azi diy ba ei esu Ms Harriet Chemusto   (telefon number 0392 174 236) ogwa ipi Mildmay Uganda Research and Ethics Committee. Ba mani vini ava onitaa diyi ma asisile ra. Ava onitaa nderi ma afa erini lele diyi dria ra. Ba omui vini zitaa mani diyi capirisi ra. Ma aira avuzo onitaa diyi ma baru. Eyo azini onita madria arimi eri esu Research and Ethics Committee(REC) ogua nyivu, nkani eyo adari madria.

Ru ba onitaa niri ………………………………………..

Dritiza maniri……………………………………………

Mba odu…………………………………………………...

Dri ago maniri……………………

Maru sadinini (Sadini ka afa sizukuria)

Dri tiza ba onita onipiri ni…………………………..

Mba odu……………………………………………..

Maru (Ba aiza onita ma baniri esupiri)………………….

Dritiza maniri
Mba odu…………………………………………………….

Appendix 2. Afa eyo onizuri karatasi alatoa ma obi niri.

Number azoba onitaa niri

Ba ofipiri ……………………………………….

Mba odi vini anguniri……………………..

Azoba acetaa

1. Ru……………………………….
2. Distrikiti…………………………
3. Nyapara(akuti)……………….
4. Telefoni ma number 1…………………………………….
5. Telefoni ma number 2……………………………………………
6. Telefoni ma number 3………………………………………
7. LC1 ma kome ripi…………………………………………….
8. LC1 ma kome ipi ma namba telefoni driri…………………
9. Azoba ma azi erini ngazi…………………………..
10. Pazi azi nga zuri (ka ovu ciria)……………………………
11. Geriko azini bani ecozu mu esuziri……………………………………..
……………………………………………………………………………..
…………………………………………………………………………….

Number azoba onitaa niri

12. Eli

13. Mi tapa sera?

1. Eh 2. Yo 3. Kuja

14a. Mi mvapie alea?

1. Eh 2. Yoo 3. Sa azinisi 4 Azini yoo

14b. Ka ovu mvapie alea , ipe tini dakitari vu.

15. Azo azi mibe okporu nici?

1. Eh 2. Yoo

16. Ka ovu ci,azo ngo ci

---

17. Mi azo sukari niri be ya?

1. Eh 2. Yoo 3. Aniku 4. Azini yoo

18. Mi azo asiazo niri be ya?

1. Eh 2. Yoo 3. Aniku 4. Azini yoo(Ipe alenia)

19. Mi azo turufuru azo iri be ya?

1. Eh 2. Yoo 3. Aniku 4. Azini yoo(Ipe alenia)

(Ngo ii)……………………………..

20. Mi azo ari ombaza iri be ya?

1. Eh 2. Yoo 3. Aniku

21. Mi azo azini econi erepiru ba ndundu vuri be ya?

1. Eh 2. Yoo 3. Aniku

22. Ka ovu ci, ngo ii?

1. Azo oguma alia B, 2. Ruri Cii, 3. Azo ondrindria, 4. Azo agale, 5 Azini yoo

23. Mi aro ti azitani bede rile ya?

1. Eh 2. Yoo 3. Azini yoo

2.

Number azoba onitaa niri ☐☐☐

24. Eka eco ra, aro ngo ii?
    1……………………………………
    2…………………………………..
    3…………………………………..
    4…………………………………

25. Ba ba mi rua operesoni sawa azisi ra ya? ☐
    1. Eh 2. Yoo 3. Aniku 4Azini yoo(Ipe alenia)

26. Odu ngo si? ☐

    1. Mba alipi bodi ma alia 2. Mba iri ma vutia 3. Aniku 4azini yoo alenia ☐

27. Eka ovu operesoni be , afa andra bani liri adu ni ya?
    1. Azo agale 2. Aniku 3. Anzini yoo(Ipe alenia)

28. Mi ogale tuu baya? ☐
    1. Arojoa 2. Nosiku arojoazi nduni ma alea 3. Clinic sendesirima alea 4.Aniku

29. Mi azo azi mi ogale aluria noku izitro woro ra ya? ☐
    1. Yoo 2. Triadaria 3. Trienjia 4. Triada nosiku trienji 5 Anzini

30. Mi suta eri pari eri maaleyaraya?
    1.Azoza yo ☐
    2. Azoza cii te mi eco eyo avii drinia ra. ☐
    3.Azoza eii , mi eco eyo, avii drinia ku tea tri minba azigaza ku.
    4.Azoza cii,mi eco eyo avii drinia ku evi vini. Mini asi bazaar azingaza driari oga si ☐
    5. Azoza cii mi eco eyo asii drinia ku eri vini mini azi ngaza oga si. ☐

Number azoba onitaa niri

6. Azoza ci, mi eco eyo avi drinia ku,eri vini le minia li aza kitanda ma dria. ☐

7.Azoza ci, mi eco eyo asi drinia ku,eri vii le I ma mu daktaria. ☐

31.Mi abi azoza mi agaleari esu edozu ngoari ya? ☐

1.Emba asia 2. Agaa emba asia 3. Aniku. ☐

32.Mi oku agale azoza esu paleko alu alu ray a?

1. Eh 2 Yoo 3. Aniku ☐

33. Eyo azii mini eco ece ece ri…………..

………………………………………………………………………..

………………………………………………………………………..

Appendix 6.

Number azoba onitaa niri

Azoba mu vuti obiza yumula iri ma vutiari

Ba ofipiri ……………………………………….

Mba odi vini anguniri……………………..

Azoba acetaa

1. Ru……………………………….
2. Distrikiti…………………………
3. Nyapara(akuti)………………….
4. Telefoni ma number 1…………………………………………...
5. Telefoni ma number 2……………………………………………
6. Telefoni ma number 3…………………………………………..
7. LC1 ma kome ripi……………………………………………….
8. LC1 ma kome ipi ma namba telefoni driri……………………..
9. Azoba ma azi erini ngazi……………………………………….
10. Pazi azi nga zuri (ka ovu ciria)…………………………………
11. Geriko azini bani ecozu mu esuziri…………………………….
   ………………………………………………………….............
   ……………………………………………………………..

Number azoba onitaa niri

12.Eyo azini onzini mi esupi operesoni hania niri ma vutia ni cii ya?

1. Eh 2. Yoo 3. Anzini yo

13.Ka ovu cii, eyo onzi ngo ii?...

1.Suta angiri lamima nki aroo suta atripiri, 2 .Ari raza imbile ma milea osiku agale ma alea,

3 .Ari raza nosiku imbile ma tusa inyiriko marua, 4. Suta aroo lepini , 5. Suta lepini bamaayu imbile ma mile, 6. Eyoo ewaru lepi mima su odre, nosiku mubira odreni suzaruni , 7. Ari olisa mimapa nosiku ongua , 8. Asoo ongua, 9. Asimaalea, 10.Hernia n indri edazu vule, 11.Azii ni.

14.Eyo azini onzini ka ovu cii, ombgnoni ngopi?

1= Were , 2= Angavusi, 3= Angiri, 4 = Drazu teru.

15.Mi api operesoni ndesira ya?

1= Eh 2= Yoo 3= Anzini yoo

16. Emu arojoa azinia, noku mi esu aro bile hania nirisi operesoni ma vutia ray a?

1= Eh 2= Yoo

17. Mi eco ai me agui noku mi ba azini ni operesoni hania vuri lizu aro bani tezu mi lizu riri ray a?

1= Eh 2 = Yoo 3. Anzini yoo (Optios).

18. Mini egarisi ba eco operesoni hania vuri edezu oduvu ray a?

1= Eh 2= Yoo 3. Anzini yoo (Options)

19. Egataa azini ndu diyi………………………………………………………………………..

Appendix 7.

Number azoba onitaa niri

Azoba ma vuti obiza eli alu ma vutiari

Ba ofipiri ……………………………………….

Mba odi vini anguniri……………………..

Azoba acetaa

1. Ru………………………………
2. Distrikiti………………………..
3. Nyapara(akuti)………………….
4. Telefoni ma number 1…………………………………………...
5. Telefoni ma number 2……………………………………………
6. Telefoni ma number 3……………………………………………
7. LC1 ma kome ripi………………………………………………..
8. LC1 ma kome ipi ma namba telefoni driri……………………….
9. Azoba ma azi erini ngazi…………………………………………
10. Pazi azi nga zuri (ka ovu ciria)…………………………………..
11. Geriko azini bani ecozu mu esuziri……………………………...
……………………………………………………………………
……………………………………………………………………

Number azoba onitaa niri

Eyoo mini eco zi sutama ndria sawa disi ni nosiku operesoni mandria wiki alipibo mandria rii.

12. Mi ama suta operesoni madriari odu alipi bondi mandria raa.

1. Azoza cii, ☐
2. Azoza cii, te mi eco eyo avii drinia ku,te econi mini mina azi ngaza ogaa ku. ☐
3. Azoza cii, te mi eco eyo avii drinia ku, te econi mini mia azi ngaza ogaa ku. ☐
4. Azoza cii, me eco eyo avii drinia ku, ere lee azi ngaza atri mini cii. ☐
5. Azoza cii, mi eco eyo avii drinia ku eri mini azi ngaza atri cii. ☐
6. Azoza cii, mi eco eyo avii drinia ku, eri lee mi ma lu ava kitada ma dria. ☐
7. Azoza cii , mi eco eyo avii drinia ku,eri lee mi ma mu daktaria. ☐

13. Mima omvitaa lo ari ka adere azoza yo,

2. Azoza avii mba alu kpere na bani operesoni adezuri ma alea ☐
3. Azoza avii mba su kpere azia bani operesoni adezuri ma alea. ☐
4. Azoza avii mba aziri kpere mudri drini iri bani operesoni adezuri ma alea. ☐
5. Azoza avi nga odiru. ☐

14. Palako si mini azoza asuzu mina agale bani lizi ma aleazi?

☐

1.Alu yumula alu alea. ☐

2.2-5 yumula alu alea. ☐

3. Odu driasi. ☐

4. Odu azini ini driasi. ☐

☐

Number azoba onitaa niri

5. Yumula alu pi.

15. Eci aro azoza atizuri azoza mini asule mima agale bani liri ma aleari atizu yumula agape dii ma alea ra ya.

1.Yoo 2. Eh 3. Anzini yoo.

16. Sawa ngopi erife mini suta ni azi ogazu minu emba eriimavuti rayaa?

1. Ma embi ezii ngazi raa.

2. Suta fe ma ambini azii ngazu ku endosu odu alu case aziri emba alipi iri dimaalea.

3. Suta ongamani azzi ngazu odu alu casu suu embaalipi iri dimaalea.

4.Suta ongamani azii ngazu emba alipi iri dimaalea.

17. Andro ,I esu ojata suta ma alea operesoni si rayaa? Suta azini onuta operesoni ndria eri alurile nosiku were.

1. Were 2. Alurile 3. Angiri

18 Mi enga operosoi vutia mukeraa?

1. Eh 2. Yoo 3 Anzini yoo

19. Ka andriuo eyo asipiri ngonyi

1. Suta 2. Dri oyaza were 3. Diridiri.

20. Miapi operasoni hania furisi sayaa?

1. Eh 2. Yoo

21. Ma embi mi asi operasoni hania ni nosiku alea operesoni hania………………………….

Aroojo eli kali iri………………………………………………………… 1.Eh 2. Yoo

Appendix 8.

Number azoba onitaa niri

Eyo aceza azoba ni operesoni ma vutiari.

Eyo were were mini eco esu operesoni ma vutia diyi.

Operesoni ma vutia, minga suta azi ra. Iborofini azini panadodi caki oni ma azoza ma suza atrizo ra. Le mima ayu asipirini ozini dikilorofenaki, denga podi imu operesonia ku, mu tezu mu ma ari ni razu ambamba ku beni.

- Suzasi eri awazu mini azi ngazu odu 10-14 operesoni ma vutia. Te eri muke mini mi rubati oyazu vini acizo driorile.
- Sa worosi ari raza were ni odre cii diyi eco mima adroo ma kala gara. Diyi ma eyo azi yo, eri nga avii odu 30 vutia.
- Nyabiliko ni nga ovu oseru vini okporu mba edozu operesoni ma vutia ria. Te were were,ngabilikori nga alea akazaru.
- Nyabiliko ma agai eco ovu diri diri ri,te diyi ma eyo yo.
- Ba nga wizi onze odu 14 ma vutia nee eyo ozini onzi mi be ciari esuzo.
- Eyo onzi bani osu operesoni ma vutia dakitari lezo ezu ani diyi.
- Azo bile ma alea diyi. Bile ni nga tutu, azirii azoazo , asiria eziri azoazo, asiria eringa era engazu aria ri si.
- Ari mbaza. Ari raza anyiriko ma etiari nga te bile noku adroo ninga ovu tuzaru.
- Eyo diiyi ka nga mi bi ra, ama ai mi Dakitari Alphonsus Matovu ni omvezu telefoni namba 0774287185, emini ecozu eyo nderi nzezuu vini ba nga odi adeari otuzu,ka ecoraria.

Eyo diyi ma atziza odu 14 ma vutiari ninga ovu……………………………. (odu) sawa …………………………. Si pari ………………………………. Aa, Dakitari ……………………………………. Ni nga mi ne ni.
