# Supplementary material for: Open Anterior Mesh Repair vs Modified Open Anterior Mesh Repair for Groin Hernia in Women: A Randomized Clinical Trial
Source: JAMA Surg. 2025 Jul 16;160(9):946–53. doi: 10.1001/jamasurg.2025.2244 (PMC12268526; doi:10.1001/jamasurg.2025.2244)
Supplement: Supplement 2. — Statistical Analysis Plan. [file jamasurg-e252244-s002.pdf]

FEMREP study. Statistical Analysis Plan(Original)

Data analysis will be performed using primarily Excel and SPSS. Counts will be presented as numbers and per cent and comparison of binary values will be done using chi square test, Fischer exact test or an exact binomial test as appropriate. Continuous data will be presented as mean and standard deviation and analysis will be done using students t-test. Absolute difference between the study groups for the primary and secondary endpoints will be calculated and presented with 95% confidence intervals. A difference of more than 5 percentage points for the primary endpoint (recurrence) is considered clinically relevant. A p-value of 0.05 is considered statistically significant.
