# Supplementary material for: Open Anterior Mesh Repair vs Modified Open Anterior Mesh Repair for Groin Hernia in Women: A Randomized Clinical Trial
Source: JAMA Surg. 2025 Jul 16;160(9):946–53. doi: 10.1001/jamasurg.2025.2244 (PMC12268526; doi:10.1001/jamasurg.2025.2244)
Supplement: Supplement 3. — eFigure. Consort Flow Chart Per-Protocol Analysis eTable 1. Intraoperative Groin Hernia Findings Among Study Participants Stratified by Allocation Arm and by Intention-to-Treat and Per-Protocol Analysis eTable 2. Recurrent Hernias in 11 Study Participants—Index Operation and Findings at Reoperation eAppendix. Participants Who Died Before the 1-Year Follow-Up eTable 3. Hernia Recurrence and Death Stratified According to the Allocation Arm, Per- Protocol Analysis [file jamasurg-e252244-s003.pdf]

Supplementary Online Content

Matovu A, Nordin P, Wladis A, et al. Open anterior mesh repair vs modified open anterior mesh repair for groin hernia in women: a randomized clinical trial. *JAMA Surg*. Published online July 16, 2025. doi:10.1001/jamasurg.2025.2244

eFigure. Consort Flow Chart Per-Protocol Analysis

eTable 1. Intraoperative Groin Hernia Findings Among Study Participants Stratified by Allocation Arm and by Intention-to-Treat and Per-Protocol Analysis

eTable 2. Recurrent Hernias in 11 Study Participants—Index Operation and Findings at Reoperation

eAppendix. Participants Who Died Before the 1-Year Follow-Up

eTable 3. Hernia Recurrence and Death Stratified According to the Allocation Arm, Per-Protocol Analysis

This supplementary material has been provided by the authors to give readers additional information about their work.

© 2025 Matovu A et al. *JAMA Surgery*.

eFigure. Consort Flow chart per-protocol analysis[a]

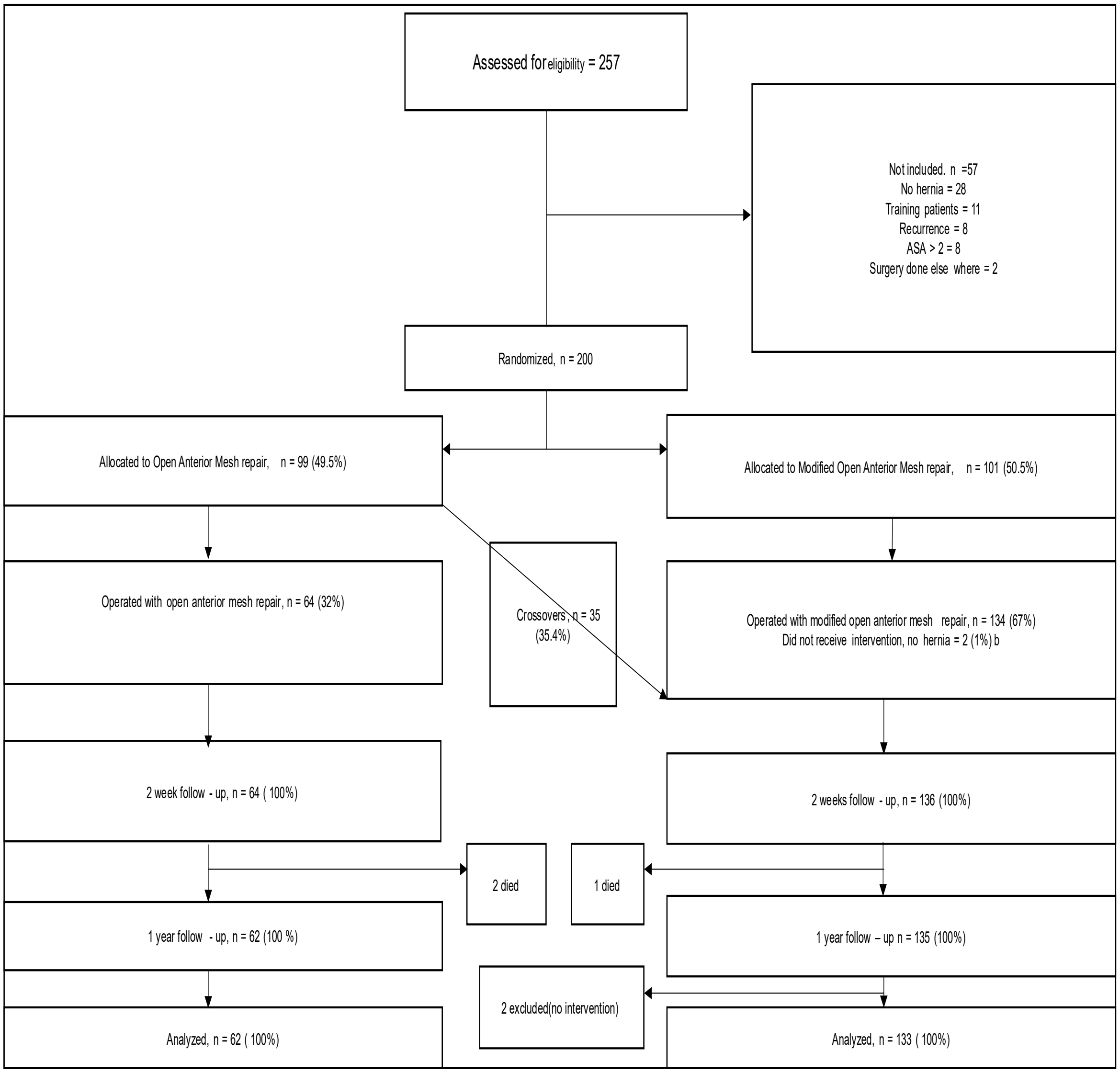

a In this flow chart, two participants in the control arm who died before the one year follow up are excluded from analysis as the outcome measures could not be assessed in those study participants. One participant in the intervention arm also died before the one year follow up.

b Two participants were excluded from the analysis because they did not have any of the interventions. One had no mesh and another had a mesh placed for a Spigelian hernia.

© 2025 Matovu A et al. *JAMA Surgery*.

eTable 1. Intraoperative groin hernia findings among study participants stratified by allocation arm and by intention – to - treat and per - protocol analysis

Hernia anatomy	Participants number, No (%)					
	Intention-to-treat analysis		P value	Per-protocol analysis		P value
	Open anterior mesh repair, n=99	Modified open anterior mesh repair, n=99[b]		Open anterior mesh repair, n=64	Modified open anterior mesh repair, n=134	
Inguinal hernia						
Medial hernia	13 (13.1)	11 (11.1)	0.66	13 (20.3)	11 (8.2)	0.02.
Lateral hernia	46 (46.5)	32 (32.3)	0.04	47 (71.9)	31 (23.1)	<0.001
Combined medial and lateral	2 (2.0)	2 (2.0)	1.0	2 (3.1)	2 (1.4)	0.45
Femoral hernia	26 (26.3)	32 (32.3)	0.35	0	58 (42.5)	<0.001
Combined anatomy						
Lateral and femoral hernia	8 (8.0)	17 (17.2)	0.05	0	25 (18.7)	<0.001
Medial and femoral hernia	2 (2.0)	4 (4.0)	0.41	0	6 (4.5)	0.09
No hernia	2 (2.0)[c]	1 (1.0)[d]	0.56	2 (3.1)[c]	1 (0.7)[d]	0.20

[b] Two participants in the modified open anterior mesh repair did not have the intervention. One had no hernia and another had a mesh placed for a Spigelian hernia. These were excluded from the analysis.
[c] Two participants with no hernia in the control arm had open anterior mesh repair. One had a large weakness in the posterior wall, and another had a wide internal ring.
[d] One participant with no hernia in the intervention arm had a modified open anterior mesh repair because of a very large weakness in the femoral canal.

© 2025 Matovu A et al. *JAMA Surgery*.

eTable 2. Recurrent hernias in 11 study participants – index operation and findings at re-operation[a]

Index operation, n (%)	Findings at re-operation	Conclusion
Open anterior mesh repair for lateral hernia, 2 (18.2)	Femoral hernia, n=2 (18.2%)	Possibly a new or missed femoral hernia
Modified open anterior mesh repair for femoral hernia, 4 (36.4)	Femoral hernia, n=4 (36.4%)	Inadequate placement of the mesh
Modified open anterior mesh repair for a femoral hernia, 4 (36.4)	Lateral hernia, n= 4 (36.4%)	Possibly a new or missed lateral hernia
Modified open anterior Mesh repair for a femoral and lateral hernia, 1(9)	Lateral hernia, n= 1 (9%)	Possibly a new lateral hernia

[a]The Nyhus technique was used for all study participants with recurrent hernia.

© 2025 Matovu A et al. *JAMA Surgery*.

eAppendix. Participants who died before the one-year follow up.

Participant 1. Was well postoperatively but later developed difficulty to swallow solid foods and subsequently liquids. She was diagnosed with esophageal cancer, and died 422 days after the hernia surgery.

Participant 2. She was well in the postoperative period. She was diagnosed with HIV and died of HIV/AIDs-related complications 468 days after the hernia surgery.

Participant 3. Was well postoperatively but then developed yellowing of the eyes and abdominal pain in the right upper quadrant. She was diagnosed with cancer of the liver and passed away 494 days after the hernia surgery.

© 2025 Matovu A et al. *JAMA Surgery*.

eTable 3. Hernia recurrence and death stratified according to the allocation arm, per- protocol analysis[a]

Outcome	Participants No./Total No (%)		Absolute difference, percentage points (95% CI)	p-value
	Open Anterior Mesh Repair	Modified Open Anterior Mesh Repair		
Recurrence	2/62(3.2)	9/133(6.7)	-3.5 (-9.7 to 2.6)	0.318
Death (PP)	2/64 (3.1)	1/134 (0.7)	2.4 (-2.1 to 6.9)	0.200

Abbreviations.CI, confidence interval

[a] The analysis for recurrence in this table is for 195 participants. Two participants were excluded from the analysis because they did not have any of the interventions and three participants had died by the time of the one year follow up leaving 195 participants for analysis.

© 2025 Matovu A et al. *JAMA Surgery*.
