# Supplementary material for: Open Anterior Mesh Repair vs Modified Open Anterior Mesh Repair for Groin Hernia in Women: A Randomized Clinical Trial
Source: JAMA Surg. 2025 Jul 16;160(9):946–53. doi: 10.1001/jamasurg.2025.2244 (PMC12268526; doi:10.1001/jamasurg.2025.2244)
Supplement: Supplement 4. — Data Sharing Statement. [file jamasurg-e252244-s004.pdf]

Data Sharing Statement

Matovu. Open Anterior Mesh Repair vs Modified Open Anterior Mesh Repair for Groin Hernia in Women. *JAMA Surg*. Published July 16, 2025. doi:10.1001/jamasurg.2025.2244

Data
Additional Information: ISRCTN 1033068, https://doi.org/10.1186/ISRCTN10330683
Data available: Yes
Data types: Deidentified participant data
How to access data: alphonsusing@gmail.com
When available: With publication

Supporting Documents
Document types: Other (please specify)
Additional Information: Trial protocol including statistical analysis plan
How to access documents: alphonsusing@gmail.com
When available: With publication

Additional Information
Who can access the data: Researchers in the field who have approval to access the data
Types of analyses: Pseudonymized data can be analyzed in accordance with what has been applied for in the ethical approval
Mechanisms of data availability: After approval of a proposal and a signed data access agreement
Any additional restrictions: Metadata will most likely not be possible to use for analysis
